# NIR-II photothermal-driven pulsatile PTH release for osteoporotic bone repair

**DOI:** 10.1093/nsr/nwag407

**Published:** 2026-07-06

**Authors:** Yiqing Wu, Yongkang Yao, Zirui He, Fangru Xie, Dongyong Sha, Chenxu Yan, Zhiqian Guo, Yuan Yuan, Wei-Hong Zhu, Changsheng Liu

**Affiliations:** Key Laboratory for Ultrafine Materials of Ministry of Education, Frontiers Science Center for Materiobiology and Dynamic Chemistry and School of Materials Science and Engineering, East China University of Science and Technology, Shanghai 200237, China; Key Laboratory for Advanced Materials and Joint International Research Laboratory of Precision Chemistry and Molecular Engineering, Shanghai Key Laboratory of Functional Materials Chemistry, Feringa Nobel Prize Scientist Joint Research Center, Institute of Fine Chemicals, Frontiers Science Center for Materiobiology and Dynamic Chemistry, School of Chemistry and Molecular Engineering, East China University of Science and Technology, Shanghai 200237, China; Shanghai Frontier Science Research Base of Optogenetic Techniques for Cell Metabolism, School of Pharmacy, East China University of Science and Technology, Shanghai 200237, China; Key Laboratory for Ultrafine Materials of Ministry of Education, Frontiers Science Center for Materiobiology and Dynamic Chemistry and School of Materials Science and Engineering, East China University of Science and Technology, Shanghai 200237, China; Key Laboratory for Ultrafine Materials of Ministry of Education, Frontiers Science Center for Materiobiology and Dynamic Chemistry and School of Materials Science and Engineering, East China University of Science and Technology, Shanghai 200237, China; Key Laboratory for Ultrafine Materials of Ministry of Education, Frontiers Science Center for Materiobiology and Dynamic Chemistry and School of Materials Science and Engineering, East China University of Science and Technology, Shanghai 200237, China; Key Laboratory for Advanced Materials and Joint International Research Laboratory of Precision Chemistry and Molecular Engineering, Shanghai Key Laboratory of Functional Materials Chemistry, Feringa Nobel Prize Scientist Joint Research Center, Institute of Fine Chemicals, Frontiers Science Center for Materiobiology and Dynamic Chemistry, School of Chemistry and Molecular Engineering, East China University of Science and Technology, Shanghai 200237, China; Key Laboratory for Advanced Materials and Joint International Research Laboratory of Precision Chemistry and Molecular Engineering, Shanghai Key Laboratory of Functional Materials Chemistry, Feringa Nobel Prize Scientist Joint Research Center, Institute of Fine Chemicals, Frontiers Science Center for Materiobiology and Dynamic Chemistry, School of Chemistry and Molecular Engineering, East China University of Science and Technology, Shanghai 200237, China; Key Laboratory for Ultrafine Materials of Ministry of Education, Frontiers Science Center for Materiobiology and Dynamic Chemistry and School of Materials Science and Engineering, East China University of Science and Technology, Shanghai 200237, China; Key Laboratory for Advanced Materials and Joint International Research Laboratory of Precision Chemistry and Molecular Engineering, Shanghai Key Laboratory of Functional Materials Chemistry, Feringa Nobel Prize Scientist Joint Research Center, Institute of Fine Chemicals, Frontiers Science Center for Materiobiology and Dynamic Chemistry, School of Chemistry and Molecular Engineering, East China University of Science and Technology, Shanghai 200237, China; State Key Laboratory of Green Chemical Engineering and Industrial Catalysis, Center of Photosensitive Chemicals Engineering, East China University of Science and Technology, Shanghai 200237, China; Key Laboratory for Ultrafine Materials of Ministry of Education, Frontiers Science Center for Materiobiology and Dynamic Chemistry and School of Materials Science and Engineering, East China University of Science and Technology, Shanghai 200237, China

**Keywords:** photothermal, photoresponsive, near-infrared, osteoporotic bone repair, NIR-II dye

## Abstract

Integrating light-controlled intelligent materials into three-dimensional (3D) printing technology holds great promise for bone tissue engineering, enabling spatiotemporal control over *in situ* drug dosing. However, this integration faces a critical molecular design challenge, as incorporated photoresponsive molecules must simultaneously fulfill the dual demands of high ultraviolet (UV)-stability during UV-mediated photopolymerization, and strong long-wavelength absorption for deep-tissue activation. Here, we report a wavelength-selective quinolinium dye (QC7CN) as photoresponsive ‘dye ink’ for addressing this dilemma. This dye features high UV transparency that ensures stability during the printing process, while possessing robust second near infrared (NIR-II) absorbance for deep-seated photothermal responsiveness. Leveraging these properties, we engineer an NIR-II light-controlled 3D-printed scaffold, composed of methacryloylated gelatin and calcium phosphate oligomers as a printing matrix, thermosensitive phase-transformable microspheres as a parathyroid hormone (PTH) drug carrier and QC7CN as a photothermal trigger. Upon NIR-II laser irradiation, QC7CN induces localized mild hyperthermia to drive the gel–sol phase transition of microspheres, thereby manipulating pulsatile PTH release for deep-tissue osteoporotic bone repair. As such, our study provides a full demonstration in molecular design and additive manufacturing to push the limits of intelligent materials in precision medicine.

## Teaser

This work develops a novel quinolinium dye as a photoresponsive ‘dye ink’, allowing for engineering a light-controlled 3D-printed scaffold to remotely manipulate pulsatile PTH release for deep-tissue osteoporotic bone repair.

## INTRODUCTION

Osteoporotic bone defects, characterized by fragility fractures from imbalanced bone resorption and formation, pose a significant clinical challenge [1]. Recently, three-dimensional (3D) printing holds immense potential in bone tissue engineering, enabling the precise fabrication of patient-specific scaffolds with complex geometries, high reproducibility and biocompatibility [2–4]. By integrating diverse bioactive factors and therapeutic drugs (proteins, peptides, amino acids and so on) [5,6], 3D printing provides a versatile platform for constructing multifunctional scaffolds that address the multifaceted demands of bone regeneration. However, a significant limitation of current functional scaffolds is their tendency to undergo uncontrolled leaching or release of bioactive compounds, attributable to their characteristic bursting effect [7]. This issue is particularly critical for drugs requiring specific release kinetics. A notable example is US Food and Drug Administration (FDA)-approved osteoporosis therapeutic agent parathyroid hormone (PTH) [8,9], whose efficacy is highly dependent on precise dosing regimens: pulsatile administration promotes osteoblast activity and bone formation, whereas continuous exposure paradoxically stimulates osteoclast-mediated resorption [10–14]. Therefore, there is an urgent demand to design an intelligent scaffold system capable of switchable, spatiotemporally controlled drug release towards bone repair.

Light, as a non-invasive external trigger, can remotely drive a closed system at high spatial and temporal precision [15–18]. By incorporating photoresponsive molecules into materials, a diverse array of light-controlled systems has been designed [19–23]. These systems can accurately identify light stimuli and elicit subsequent changes in properties, such as conformational switches [24,25] and shape/phase transformations [26]. However, developing such light-controlled scaffolds for bone repair remains challenging, as it requires incorporated photoresponsive molecules to not only be compatible with printing fabrication conditions but also overcome tissue penetration barriers: (i) ultraviolet (UV) stability during fabrication. The widely used polymerization-based 3D printing predominantly employs high-energy UV light to activate photoinitiators for monomer crosslinking and solidification [27]; thus, photoresponsive molecules must resist prolonged UV exposure without degradation. (ii) Long-wavelength absorption for deep-tissue activation. Compared to classical visible and first near-infrared windows, the second near-infrared region (NIR-II, 900–1700 nm) offers reduced photon scattering and higher maximum permissible exposure (MPE; 1.00 W cm^−2^ at 1064 nm vs 0.33 W cm^−2^ at 808 nm) [28–33], enabling deep-tissue penetration. Unfortunately, long-wavelength molecules inherently possess extended electron delocalization and narrow energy gaps, rendering them highly susceptible to UV light [34,35]. This fundamental trade-off between long-wavelength absorption and UV stability critically restricts the construction of light-controlled 3D-printed scaffolds.

**Figure 1.**
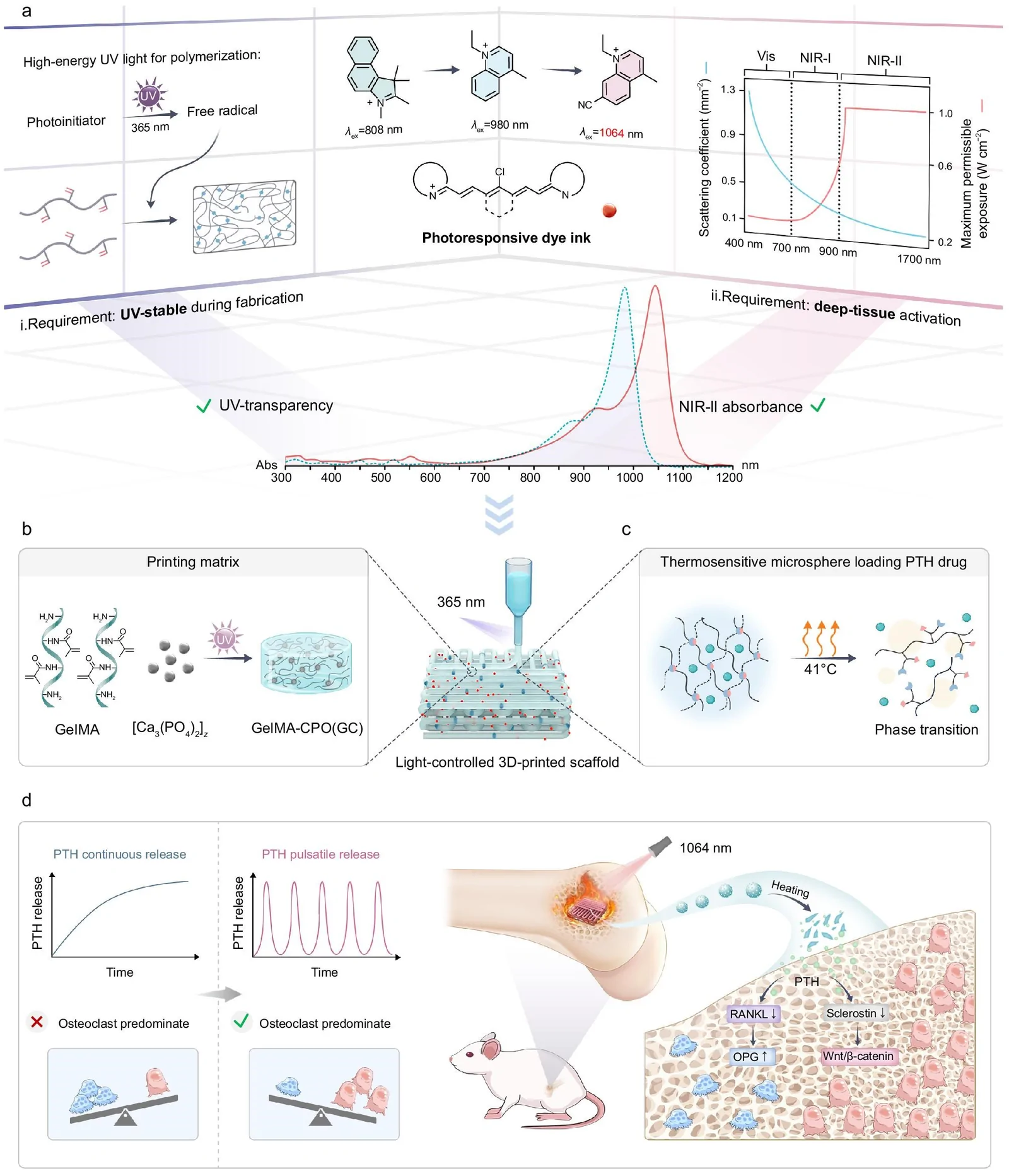
Light-controlled 3D-printed scaffold for remotely manipulating drug release for deep-tissue osteoporotic bone repair. (a) UV-transparent NIR-II quinolinium dye was designed as the printing ink, with high UV stability for photopolymerization 3D printing and strong NIR-II absorbance for deep-tissue photothermal responsiveness. (b) GelMA and CPOs were utilized as the printing matrix for high-energy UV-mediated photopolymerization. (c) Thermosensitive phase-transformable microspheres were utilized as the PTH drug carrier. (d) NIR-II photothermal-triggered pulsatile PTH release for deep-tissue osteoporotic bone repair. Abs, absorbance.

In this regard, we envisioned solving this dilemma through a paradigm shift: making photoresponsive molecules ‘invisible’ to UV light rather than ‘resistant’ to it. Since photodegradation is an absorption-initiated process, eliminating UV absorption intrinsically blocks electronic excitation and subsequent degradation pathways. Herein, we rationally designed a wavelength-selective quinolinium dye, QC7CN, which demonstrated high transparency to UV light while exhibiting strong absorption in the NIR-II region (Fig. 1). Thus, QC7CN exhibited remarkable UV-stability under continuous UV irradiation, enabling seamless integration into photopolymerization 3D printing. Meanwhile, QC7CN showed excellent NIR-II photothermal responsiveness under 1064 nm laser irradiation, achieving a photothermal conversion efficiency of 49.3%. Building upon these properties, we next engineered a light-controlled 3D-printed scaffold. This scaffold utilized methacryloylated gelatin (GelMA) and calcium phosphate oligomers (CPOs) as a photopolymerization printing matrix, thermosensitive phase-transformable microspheres as a PTH drug carrier and QC7CN as a photothermal-responsive trigger. Upon 1064 nm laser irradiation, QC7CN induced localized mild hyperthermia (∼41°C), which drives the gel–sol phase transition of the microspheres, leading to PTH on-demand pulsatile release. This controlled scaffold effectively modulates the osteogenic–osteoclastic balance, thereby promoting the repair of osteoporotic bone defects.

## RESULTS AND DISCUSSION

### UV-transparent NIR-II quinolinium cyanine dye with high UV stability

Heptamethine cyanine dyes are considered promising candidates for photoresponsive applications due to their long-wavelength absorption bands and high molar extinction coefficients [36–39], exemplified by the FDA-approved indocyanine green (ICG). Nonetheless, traditional heptamethine cyanines predominantly absorb within the 785–810 nm range, falling short of the desired NIR-II window [40]. Consequently, there is an urgent need to develop heptamethine cyanine dyes capable of absorbing in the NIR-II region to further advance *in vivo* phototherapy technologies.

**Figure 2.**
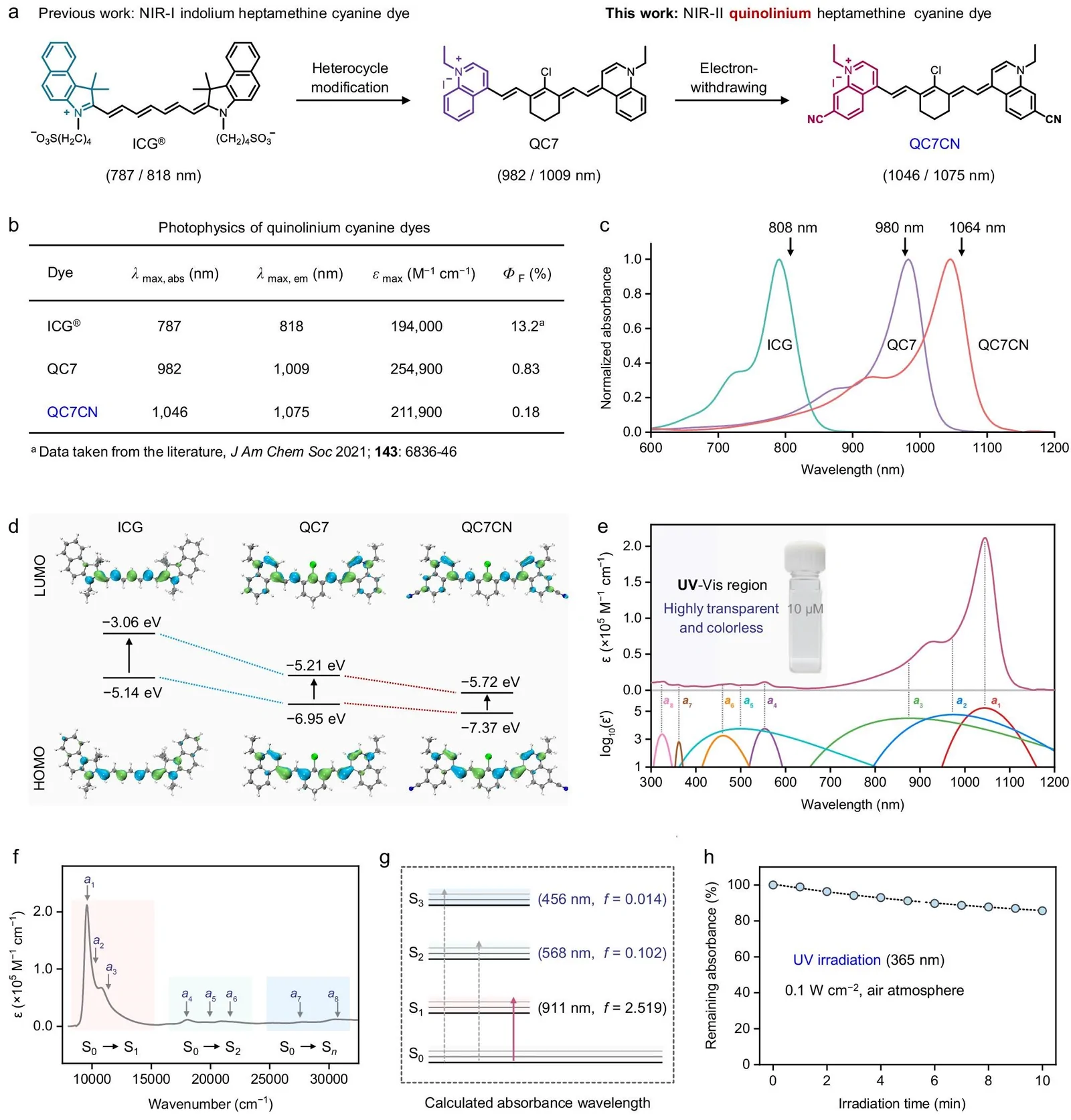
Engineering UV-stable NIR-II quinolinium cyanine dyes. (a) Molecular design of NIR-II quinolinium heptamethine cyanine dye QC7CN. (b) Table about photophysical properties of ICG, QC7 and QC7CN dyes. (c) Normalized absorption spectra of cyanine dyes across the commercial laser (λ_ex_ = 808, 980 and 1064 nm). (d) HOMO (bottom) and LUMO (top) orbital density plots of ICG, QC7 and QC7CN. (e) Absorption spectrum and corresponding deconvoluted absorption spectra of QC7CN dissolved in dichloromethane. (f) Wavenumber of the deconvoluted absorption peak of QC7CN. (g) Calculated absorption wavelengths and corresponding oscillator strength (*f*) of QC7CN. (h) Photostability of QC7CN under ultraviolet (UV, 365 nm) light irradiation.

With this in mind, we adopted a ‘step by step’ molecular design strategy to bathochromically shift the absorption wavelength of cyanine dyes (Fig. 2a and Fig. S1): (i) In ICG, the nitrogen atom on the five-membered indolium ring is bonded to the heptamethine chain via an adjacent carbon atom. In our study, we replaced the indolium unit with a quinolinium scaffold core, thereby connecting the nitrogen atom to the heptamethine chain through the para-position carbon atom. This modification resulted in the quinolinium cyanine dye (QC7), which exhibits a significant redshift of ∼200 nm compared to ICG dye. (ii) To further expand the dye’s π-conjugated system, we introduced an electron-withdrawing cyano group at the 7-position of the quinolinium core. The resulting QC7CN cyanine dye exhibited a maximum absorption wavelength from 983 to 1046 nm, which matches well with the commercial 1064 nm laser (Fig. 2b and c). To further elucidate the structure–property relationship, we performed density functional theory calculations. The energy gaps between the highest occupied molecular orbital (HOMO) and the lowest unoccupied molecular orbital (LUMO) for ICG, QC7 and QC7CN were 2.08, 1.74 and 1.65 eV, respectively (Fig. 2d). This sequential decrease in energy gap suggests an enhancement in electronic delocalization along π-conjugated molecular backbone, accounting for the observed bathochromic shift towards longer wavelengths.

We next focused our investigation on QC7CN due to its optimal absorption characteristics in NIR-II region. The absorption spectrum of QC7CN displays typical cyanine-type features, characterized by an intense and sharp long-wavelength absorption peak at 1046 nm (*ε* = 211 900 M^−1^ cm^−1^), accompanied by a shoulder peak at 927 nm (Fig. 2e). To achieve a more comprehensive understanding of the electronic transitions, the spectrum was deconvoluted into eight constituent Gaussian peaks (*a*_1_–*a*_8_) through mathematical fitting [41]. The peaks *a*_1_–*a*_3_ in the NIR-I to NIR-II spectral region (700–1100 nm) primarily correspond to the HOMO–LUMO transition from the S_0_ ground state to the S_1_ excited state, while peaks *a*_4_–*a*_8_ correspond to vertical excitations to higher excited states (S_0_ to S*_n_*) (Fig. 2f). Notably, these higher energy transitions result in only a residual broad and extremely weak absorption in the ultraviolet-visible (UV-Vis) region, endowing the dye with exceptional transparency in this spectral window. Theoretical calculations support this observation: the oscillator strength (*f*) values at 456 and 568 nm are merely 0.014 and 0.102, respectively, which are significantly lower than the value of 2.519 at 911 nm (Fig. 2g). This selective absorption profile is particularly beneficial for applications necessitating UV-Vis light transparency while maintaining strong NIR-II absorption.

These distinctive optical properties of QC7CN make it exceptionally suitable for common photopolymerization 3D printing applications. Traditional photopolymer systems employ high-energy UV light (365–405 nm) to activate radical or cation photoinitiators, and then trigger monomer crosslinking and solidification; this process often induces the photodegradation of long-wavelength dyes. Given the negligible UV absorption coefficient of QC7CN, we hypothesized that QC7CN would exhibit excellent photostability under 3D printing conditions. To validate this, we conducted photostability tests using 365 nm irradiation, a wavelength commonly utilized in commercial 3D printing instrument sets. Under continuous irradiation at 0.1 W cm^−2^ for 10 min, QC7CN retained over 85% of its initial absorption intensity (Fig. 2h). This remarkable UV stability, coupled with strong NIR-II absorption, indicates that QC7CN can function as an ideal NIR-II ‘dye ink’ in photopolymerization 3D printing.

### NIR-II photothermal responsiveness with deep-tissue penetration

**Figure 3.**
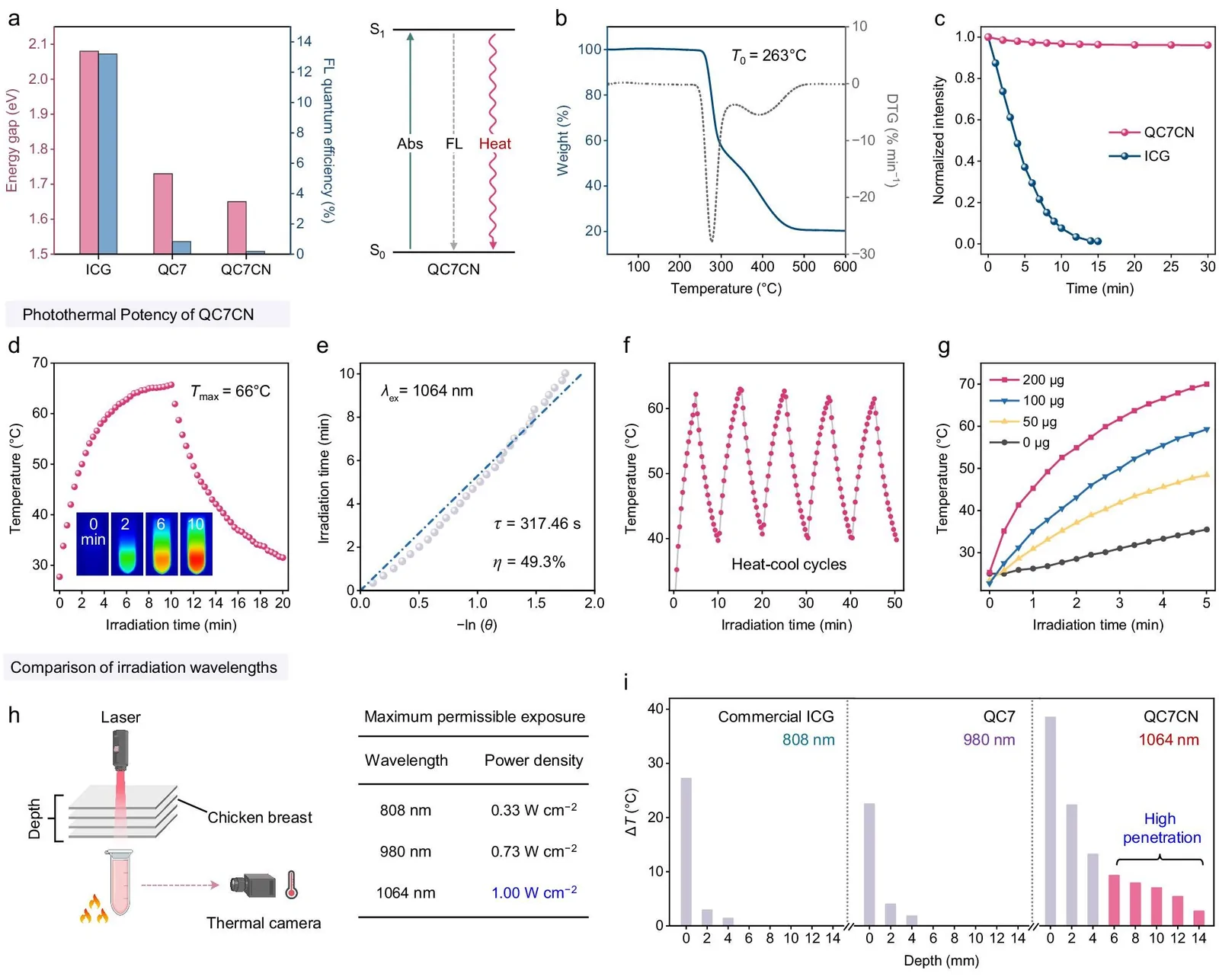
Photothermal potency and penetration depth of QC7CN. (a) Energy gap and fluorescence quantum efficiency of ICG, QC7 and QC7CN. (b) Thermogravimetry analysis of QC7CN. (c) Photostability of ICG and QC7CN under laser exposure (1.00 W cm^−2^), with excitation at 808 nm for ICG and 1064 nm for QC7CN. (d) Photothermal potency and photothermal images (inset) of QC7CN micelles in H_2_O under 1064 nm laser irradiation (1.00 W cm^−2^). (e) Linear fitting curve of the cooling time of QC7CN micelles as a function of the negative logarithm of temperature. (f) Photothermal stability study of QC7CN micelles during five cycles of heating–cooling processes. (g) Photothermal potency of QC7CN micelles at different concentrations. (h) Schematic illustration of comparative tissue penetration experiment and MPE limits. (i) Photothermal penetration depth comparison among commercial ICG (808 nm), QC7 (980 nm) and QC7CN (1064 nm) under respective MPE conditions. FL, fluorescence.

In theoretical terms, fluorescence emission (radiative decay) and heat production (non-radiative decay) in organic dyes are characterized by a competitive trade-off effect [42–44]. Efficient photothermal-responsive agents rely on maximizing non-radiative decay pathways [45–49], thereby converting absorbed photon energy into heat rather than fluorescence emission. As illustrated in Fig. 3a, QC7CN exhibited the lowest optical energy gap (1.65 eV) and fluorescence quantum yield (0.18%) among the dyes tested, indicating its potential as an exceptional photothermal-responsive agent (Fig. S2). Thermogravimetric analysis revealed that QC7CN preserved its structural integrity with a decomposition temperature of 263°C (Fig. 3b and Fig. S3), which significantly exceeds the physiological requirements for photothermal applications. Additionally, QC7CN exhibited enhanced resistance to photobleaching compared to ICG, maintaining over 95% of its initial absorption after 30 min of 1064 nm laser irradiation (Fig. 3c).

To improve biocompatibility and aqueous dispersibility, QC7CN was encapsulated within the DSPE-mPEG2000 polymer (Fig. S4). Upon 1064 nm laser irradiation at 1.0 W cm^−2^, the QC7CN micelles achieved rapid temperature elevation, reaching a maximum temperature of 66°C (Δ*T* = 38°C) (Fig. 3d). Through linear fitting analysis of the cooling curve, the photothermal conversion efficiency (*η*) was calculated to be 49.3% (Fig. 3e), which exceeds that of most reported photothermal agents. Additionally, QC7CN micelles displayed remarkable stability over five consecutive heating–cooling cycles, with negligible variation in maximum temperature elevation (Fig. 3f and g). This excellent photothermal performance suggests its potential for sustained cyclic heating applications *in vivo*.

The clinical application of photothermal-responsive agents critically depends on their ability to achieve therapeutic temperatures in deep-tissue layers while complying with laser safety standards. In this study, we evaluated the tissue penetration capabilities of ICG, QC7 and QC7CN under their respective laser wavelengths, utilizing power densities at the MPE limits [50]: 0.33 W cm^−2^ for 808 nm, 0.73 W cm^−2^ for 980 nm and 1.00 W cm^−2^ for 1064 nm (Fig. 3h). Employing chicken breast tissue as a biological model, both ICG and QC7 displayed minimal heating beyond a depth of 4 mm (the limited performance of QC7 is likely due to the strong water absorption band between 950 and 1000 nm, which significantly attenuates the transmission of 980 nm light [51]) (Fig. 3i). In contrast, QC7CN exhibited superior deep-tissue photothermal performance, achieving a temperature increase of ∼6°C at depths exceeding 12 mm. Collectively, these findings establish QC7CN as an ideal NIR-II photothermal-responsive agent, characterized by excellent stability, efficiency and deep-tissue penetration, for *in vivo* applications.

### NIR-II photothermal ‘dye ink’ integrated into 3D printed scaffolds for remote PTH release

**Figure 4.**
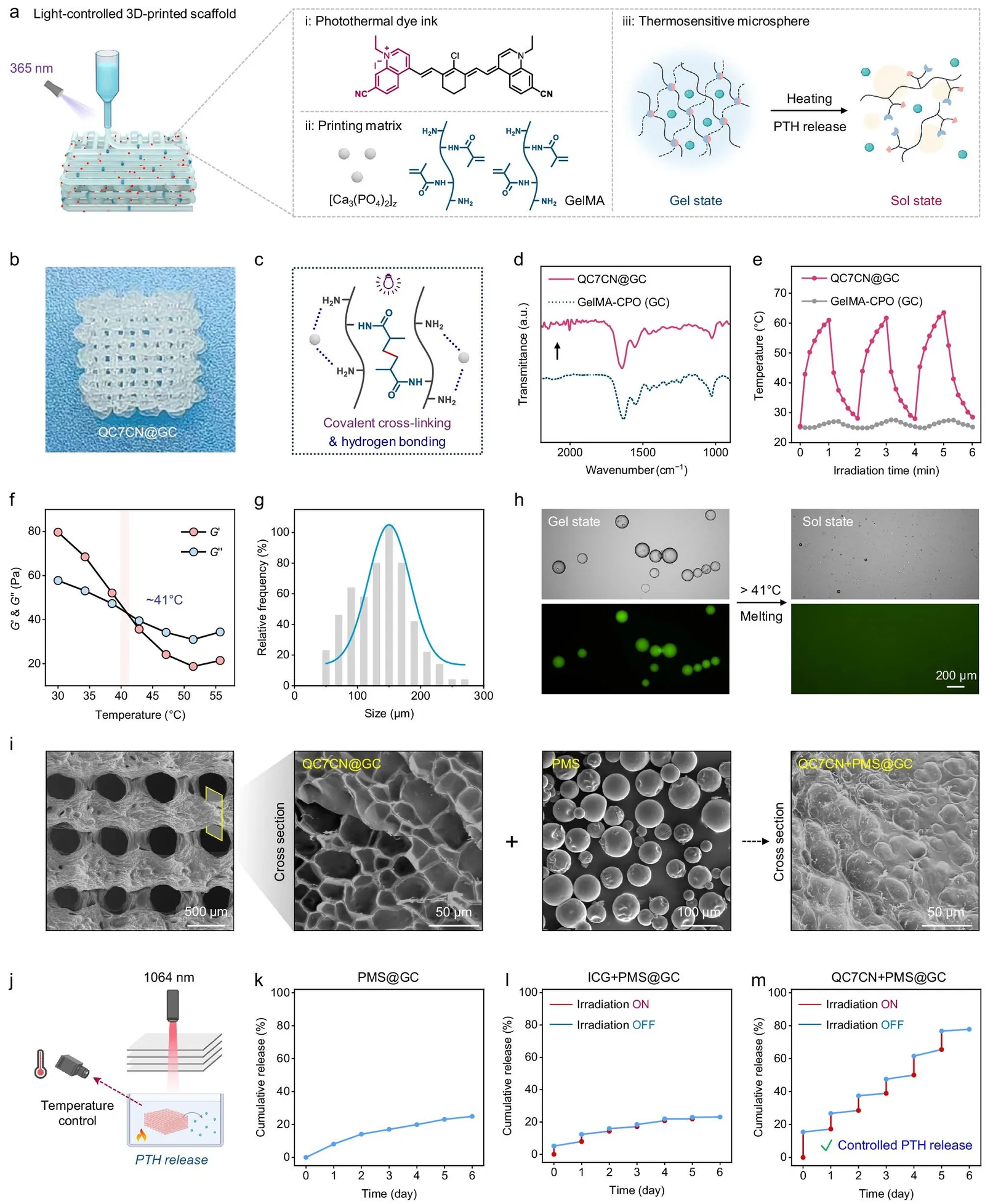
Thermosensitive microsphere integration for phototriggered PTH release. (a) Schematic illustration of the photothermal-responsive 3D-printed scaffold. (b) Photo-image of the 3D-printed QC7CN@GC scaffold. (c) Dual crosslinking mechanism in GC scaffolds: UV-induced covalent crosslinking of methacrylate groups (left), and hydrogen bonding interactions between CPO and amide groups of the GelMA backbone (right). (d) Fourier transform infrared spectra. (e) Photothermal potency of QC7CN@GC. (f) Temperature dependence of storage modulus (*G*′) and loss modulus (*G*″) for thermosensitive PNAm polymer. (g) Size distribution of thermosensitive microspheres PMS. (h) Confocal microscopy images of temperature-triggered release behavior. Calcein-encapsulated microspheres in gel state (<41°C) retain fluorescence (green), whereas heating (>41°C) induces melting and calcein release. (i) Scanning electron microscopy images of 3D-printed scaffold QC7CN@GC, thermosensitive microsphere PMS and the cross section of QC7CN+PMS@GC. (j) Schematic workflow of phototriggered PTH release assay. Scaffolds were placed beneath chicken breast tissue (5 mm thickness) to simulate deep-tissue conditions. (k–m) PTH release profiles of PMS@GC, ICG+PMS@GC and QC7CN+PMS@GC under laser irradiation.

3D printing has emerged as a transformative technology in the field of bone tissue engineering, facilitating the precise fabrication of scaffolds with intricate architectures and patient-specific geometries [52,53]. In particular, photopolymerization 3D printing technology, owing to its mild curing conditions and higher printing precision, enables the incorporation of bioactive factors and precise filling of bone defects. Leveraging the exceptional photothermal-responsive properties of QC7CN, we envision designing photothermal-responsive 3D-printed scaffolds to enable localized deep-tissue heating for drug release and bone repair (Fig. 4a).

As shown in Fig. 4b and c, GelMA and CPOs ([Ca_3_(PO_4_)_2_]*_z_*) [54,55] were utilized as the photopolymerization printing matrix (Fig. S5). In contrast to traditional calcium phosphate particles (*β*-TCP, CPC and HAp), which often aggregate and compromise scaffold integrity, ultrafine CPO can form organic–inorganic continuous structures through hydrogen bonding and charge interactions with GelMA. This molecular-level integration effectively addresses the balance between printability and bioactive mineralization capacity (Figs S6–S8). Subsequently, QC7CN was incorporated as a photothermal-responsive ‘dye ink’ into the 3D printing fabrication, as confirmed by Fourier transform infrared spectroscopy (Fig. 4d). Importantly, the high UV transparency of QC7CN ensured photochemical stability during the UV-mediated (365 nm) crosslinking process. Thus, the QC7CN@GC scaffold exhibited superior heating capabilities under 1064 nm irradiation, achieving temperatures exceeding 60°C and maintaining remarkable stability over three consecutive heating–cooling cycles (Fig. 4e and Figs S9 and S10).

To further enhance the therapeutic function of our photothermal scaffolds, we incorporated thermosensitive microspheres as drug carriers, establishing a photothermal-responsive platform for remotely controlled drug release. For the fabrication of thermosensitive microspheres, we synthesized a copolymer (PNAm) of *N*-acryloyl glycinamide (NAGA) and acrylamide (AAm) to serve as the microsphere matrix (Fig. S11). The thermal-responsive behavior of PNAm arises from the formation and disruption of intra- and intermolecular hydrogen bonds: at lower temperatures, hydrogen bonding stabilizes the polymer network in a gel state, whereas heating disrupts these interactions, triggering a gel–sol transition. Through systematic rheological screening of polymers with varying NAGA/AAm ratios, we identified an optimal formulation exhibiting a phase transition temperature of ∼41°C (Fig. 4f and Fig. S12). This temperature enables the polymer to maintain structural stability under physiological conditions (Fig. S13), while undergoing rapid gel–sol transformation upon mild photothermal stimulation.

Next, PTH-carried thermosensitive microspheres (PMS) were constructed via a modified water-in-oil emulsion-based method. The particle size was optimized to ∼140 μm (Fig. 4g). To visually observe the temperature-triggered release behavior, we employed fluorescent calcein encapsulated in microspheres as a PTH surrogate. Confocal microscopy images revealed that calcein maintained stable retention within microspheres at temperatures below 41°C, while heating above the phase transition temperature triggered rapid microsphere melting and calcein release (Fig. 4h). Subsequently, PMS were incorporated into the QC7CN@GC scaffold, designated as QC7CN+PMS@GC (Fig. S14). Scanning electron microscopy images confirmed the successful integration of PMS throughout the scaffold, with microspheres embedded within the honeycomb-like porous structure (Fig. 4i and Fig. S15).

In order to evaluate the phototriggered drug release performance, we conducted systematic *in vitro* studies using chicken breast tissue as a barrier to simulate deep-tissue conditions (Fig. 4j). As quantified by enzyme-linked immunosorbent assay (ELISA), the QC7CN+PMS@GC scaffold showed effective photothermal-triggered PTH release under NIR-II 1064 nm irradiation (Fig. 4k–m). Each pulsed irradiation induced burst release of PTH, achieving stepwise cumulative release exceeding 80% over six consecutive cycles (Figs S16 and S17). In contrast, the control scaffold without photothermal dye (PMS@GC) exhibited only passive diffusion-dominated continuous release, with cumulative PTH levels <25% over the same period. ICG-based scaffold (ICG+PMS@GC) under 808 nm irradiation showed negligible on-demand release due to low tissue penetration, highlighting the critical importance of NIR-II photothermal conversion for practical therapeutic applications.

### Modulating osteogenic–osteoclastic balance via phototriggered pulsatile PTH release

**Figure 5.**
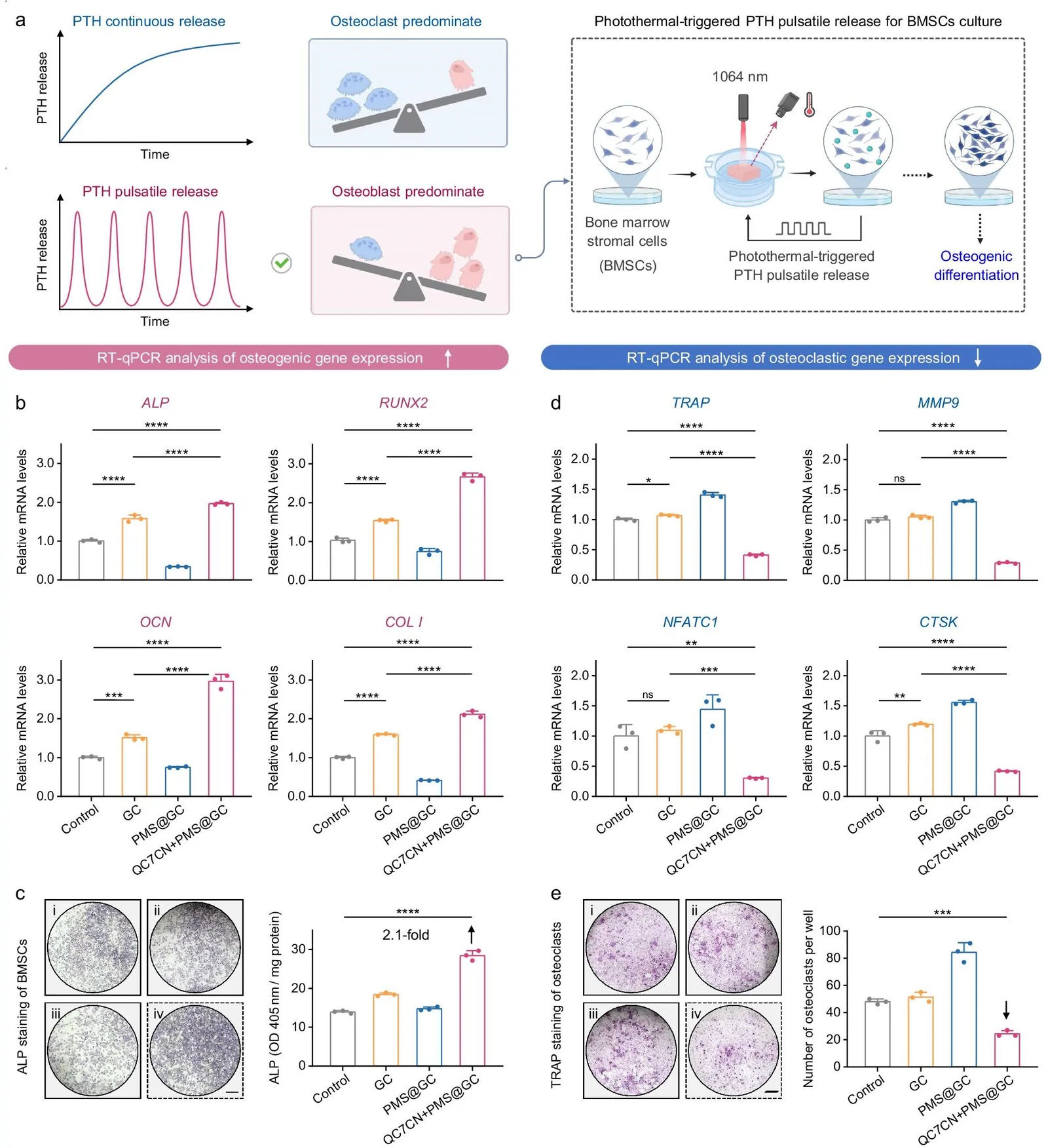
Phototriggered pulsatile PTH release promotes osteogenic differentiation while suppressing osteoclastic activity. (a) Schematic illustration showing the different effects of continuous versus pulsatile PTH release on osteoblast–osteoclast balance (left), and the experimental design for photothermal-triggered PTH release osteogenic differentiation of BMSCs *in vitro* (right). (b) RT-qPCR analysis of osteogenic marker genes (*ALP, RUNX2, OCN* and *COL I*) in BMSCs after different treatments for 3 days. (c) ALP staining and quantitative analysis of BMSCs after co-culture in an osteogenic matrix for 14 days. Scale bar: 2 mm. (d) RT-qPCR analysis of osteoclastic marker genes (*TRAP, MMP9, NFATC1* and *CTSK*) in osteoclasts after different treatments for 3 days. (e) TRAP staining and quantitative analysis of osteoclasts after different treatments for 3 days. Scale bar: 2 mm. i: control; ii: GC; iii: PMS@GC; iv: QC7CN+PMS@GC. Data are presented as mean ± standard deviation (*n* = 3). Statistical significance: *P < 0.05, **P < 0.01, ***P < 0.001, ****P < 0.0001; ns, not significant.

PTH administration has been clinically demonstrated to enhance bone mineral density (BMD), improve trabecular microarchitecture and accelerate fracture healing in osteoporosis and bone regeneration applications. However, the efficacy of PTH is highly dependent on precise dosing regimens. Specifically, continuous exposure to PTH leads to sustained activation of the PTH1 receptor (PTH1R), which upregulates the expression of receptor activator of nuclear factor kappa-B ligand (RANKL), thereby inducing osteoclast differentiation and excessive bone resorption. In contrast, pulsatile PTH release triggers transient PTH1R activation that suppresses sclerostin expression and activates the Wnt/*β*-catenin signaling pathway, preferentially promoting osteoblast differentiation and bone formation [10,56]. This dosing-dependent osteoblast–osteoclast balance highlights the critical need for smart release systems that enable on-demand, pulsatile PTH delivery at the bone defect site (Fig. 5a).

With this in mind, we performed systemic biological evaluations to validate whether our photothermal-responsive scaffold QC7CN+PMS@GC could achieve this beneficial pulsatile release pattern. Bone marrow-derived mesenchymal stem cells (BMSCs) were cultured with the scaffolds (Figs S18 and S19). Under pulsed 1064 nm laser irradiation, QC7CN+PMS@GC demonstrated remarkable photoresponsiveness, with upregulated expression of key osteogenic markers including alkaline phosphatase (*ALP*), Runt-related transcription factor 2 (*RUNX2*), osteocalcin (*OCN*) and type I collagen (*COL I*) compared to both control and GelMA-CPO (GC) groups (Fig. 5b and Table S1). In contrast, PMS@GC scaffolds exhibited markedly lower osteogenic gene expression owing to continuous PTH release, consistent with the detrimental effects described above. ALP staining and alizarin red S staining further supported these findings, with the QC7CN+PMS@GC group displaying 2.1- and 3.1-fold increases in staining intensity relative to control groups, respectively (Fig. 5c and Figs S20–S22).

Next, we evaluated the effect of QC7CN+PMS@GC on osteoclast regulation. Using osteoclasts induced from rat bone marrow macrophages as a cell model, reverse transcription quantitative polymerase chain reaction (RT-qPCR) analysis revealed that QC7CN+PMS@GC effectively suppressed the expression of osteoclast-related genes, including tartrate-resistant acid phosphatase (*TRAP*), matrix metallopeptidase 9 (*MMP9*), nuclear factor of activated T cells 1 (*NFATC1*) and cathepsin K (*CTSK*), to only 0.2- to 0.3-fold relative to control groups (Fig. 5d). In contrast, the PMS@GC group exhibited notable upregulation of these osteoclast-related genes. Consistent with the gene expression data, TRAP staining showed reduced osteoclast formation and number in the QC7CN+PMS@GC group compared to all groups (Fig. 5e). Taken together, these results highlighted that QC7CN+PMS@GC achieves dual therapeutic benefits through photothermal-triggered pulsatile PTH release: promoting osteogenic differentiation while simultaneously inhibiting osteoclast activity. This underscored the advantages of spatiotemporally controlled delivery systems for modulating osteogenic–osteoclastic balance in bone regeneration.

### Deep-tissue pulsatile PTH delivery promoting osteoporotic bone repair

**Figure 6.**
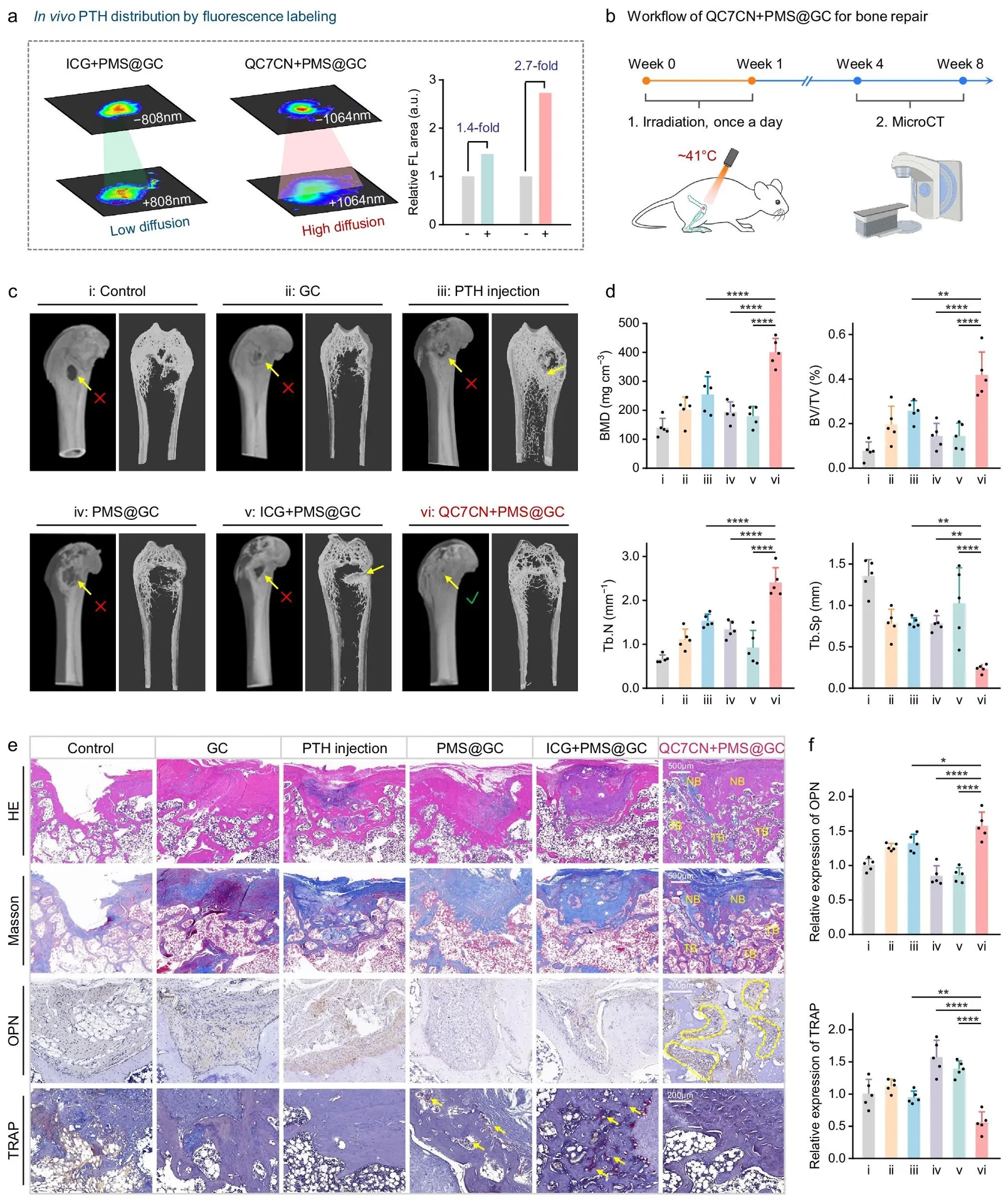
Phototriggered pulsatile PTH release for deep-tissue osteoporotic bone repair. (a) *In vivo* fluorescence images and quantitative analysis of the PTH distribution area of ICG+PMS@GC and QC7CN+PMS@GC at the defect site. (b) Schematic illustration of the experimental timeline. Rats were subjected to daily irradiation, and bone regeneration was monitored via micro-computed tomography (CT). (c) Representative 3D reconstructed micro-CT images across different treatment groups at 8 weeks. (d) Quantification of BMD, BV/TV, Tb.N and Tb.Sp. (e) HE and Masson staining, and immunohistochemical staining for OPN (osteogenesis marker) and TRAP (osteoclastogenesis marker) of femoral defect sections. (f) Quantitative analysis of OPN expression and TRAP expression at week 8. Statistical significance: *P < 0.05, **P < 0.01, ***P < 0.001, ****P < 0.0001; ns, not significant.

To validate the deep-tissue therapeutic efficacy of the QC7CN+PMS@GC system *in vivo*, we employed a rat femoral defect model established in ovariectomized (OVX) osteoporotic rats. Unlike superficial defect models, the femur site is shielded by a thick muscle layer, creating a rigorous physiological barrier to test the deep-tissue photothermal responsiveness of our system. First, to visualize *in situ* PTH release, PTH was conjugated with a Cy5.5 fluorophore (PTH-Cy5.5). As illustrated in Fig. 6a, the ICG+PMS@GC group showed minimal fluorescence distribution under 808 nm irradiation, indicating that NIR-I light could not overcome the tissue depth to trigger PTH release, with the majority of PTH retained within the scaffold. Conversely, QC7CN+PMS@GC displayed a ∼2.7-fold expansion in the fluorescent area under 1064 nm irradiation. This confirms that NIR-II light effectively penetrated deep muscle tissue to trigger the PMS phase transition, enabling the controlled PTH release.

Subsequently, the osteogenic potential of QC7CN+PMS@GC was evaluated over an 8-week period in OVX rats. During the first week post-surgery, the defect site was exposed to daily NIR-II irradiation (1 min per day), with the localized temperature maintained at ∼41°C to trigger PMS phase transition (Fig. 6b and Fig. S19). As shown in Fig. 6c, the control, GC, PMS@GC and ICG+PMS@GC groups exhibited poor bone formation, with persistent defect voids observed at week 8. In contrast, the QC7CN+PMS@GC group displayed robust osteogenesis with rapid bone bridging. The QC7CN+PMS@GC group acquired a remarkable recovery in BMD and bone volume fraction (BV/TV) (Fig. 6d and Fig. S23). Notably, at week 8, the QC7CN+PMS@GC group achieved a BMD of ∼400 mg cm^−3^, a value comparable to that of healthy, non-osteoporotic bone tissue. Moreover, the trabecular microarchitecture was significantly restored, evidenced by increased trabecular number (Tb.N) and decreased trabecular separation (Tb.Sp).

Histological assessment via hematoxylin and eosin (HE) and Masson staining (Fig. 6e) further confirmed the superior quality of bone repair. The QC7CN+PMS@GC group exhibited a significantly great abundance of trabecular bone and compact new bone. Haversian systems were clearly discernible within the lamellar bone, indicating a high degree of maturity in the newly formed bone tissue. In addition, we performed immunohistochemical staining for key markers: osteopontin (OPN, an osteogenesis marker) and TRAP (an osteoclastogenesis marker) (Fig. 6e and Fig. S23). As observed, the QC7CN+PMS@GC group exhibited upregulated OPN expression and reduced TRAP expression. Specifically, compared to the control group, OPN levels in the QC7CN+PMS@GC group increased by ∼40% at week 4 and 70% at week 8, and TRAP levels were suppressed to ∼50% (Fig. 6f and Fig. S23). These results further indicated that pulsatile PTH delivery could promote osteogenesis while inhibiting osteoclast activity. Taken together, the QC7CN+PMS@GC scaffold achieved effective, deep-tissue photoresponsiveness and remotely controlled PTH delivery, thus realizing restored bone mass, enhanced bone quality and balanced bone remodeling towards osteoporotic bone defects.

## CONCLUSION

This work demonstrates a dye design advance to develop a novel quinolinium dye as a photoresponsive ‘dye ink’. This dye enables integration into 3D printing, allowing for engineering a light-controlled 3D-printed scaffold to remotely manipulate pulsatile PTH release for deep-tissue osteoporotic bone repair. Making full use of heterocycle modification and electron-withdrawing substitution, we successfully synthesized a spectrally specific quinolinium dye QC7CN, which exhibited strong NIR-II absorption yet high UV transparency. Specifically, QC7CN retained >85% of its initial absorption intensity after continuous UV irradiation, enabling seamless integration into common photopolymerization 3D printing. Meanwhile, QC7CN showed a remarkable NIR-II photothermal conversion efficiency of 49.3% under 1064 nm laser irradiation. Building upon these advantages, we engineered a photothermal-responsive 3D-printed scaffold. Upon laser irradiation, localized mild hyperthermia triggers the phase transition of internal thermosensitive microspheres, facilitating pulsatile PTH release. *In vivo* studies in a femur defect model demonstrated that this scaffold effectively modulated the osteogenic–osteoclastic balance, significantly enhancing bone regeneration and microarchitecture restoration. Looking forward, this light-controlled design strategy holds great promise for clinical bone repair. We envision that our NIR-II platform could enable non-invasive treatments for accessible defects (e.g., knee, calvaria and tibia), while future integration with minimally invasive optical fiber catheters could seamlessly translate this strategy to deeper bone tissues.

## MATERIALS AND METHODS

Detailed materials and methods can be found in the online supplementary data.

### Ethical statements

All animal experiments in this study were reviewed and approved by the relevant Animal Ethics Committee, with the approval number ECUST-2025-200. All procedures were conducted in accordance with institutional guidelines for the care and use of laboratory animals and complied with relevant animal welfare regulations. Efforts were made to minimize animal suffering, stress, and the number of animals used. All animal housing, handling, and experimental procedures were performed according to the approved protocol.

## Supplementary Material

nwag407_Supplemental_File
