# Supplementary material for: NIR-II photothermal-driven pulsatile PTH release for osteoporotic bone repair
Source: Natl Sci Rev. 2026 Jul 6;13(17):nwag407. doi: 10.1093/nsr/nwag407 (PMC13587092; doi:10.1093/nsr/nwag407)
Supplement: Supplementary file 1 [file nwag407_supplemental_file.pdf]

NIR-II photothermal-driven pulsatile PTH release for osteoporotic bone repair

Yiqing Wu[1,†], Yongkang Yao[2,†], Zirui He[1], Fangru Xie[1], Dongyong Sha[1], Chenxu Yan[2], Zhiqian Guo[2,*], Yuan Yuan[1,*], Wei-Hong Zhu[1,3] and Changsheng Liu[1,*]

Affiliations:

[1]Key Laboratory for Ultrafine Materials of Ministry of Education, Frontiers Science Center for Materiobiology and Dynamic Chemistry and School of Materials Science and Engineering, East China University of Science and Technology, Shanghai 200237, China.

[2]Key Laboratory for Advanced Materials and Joint International Research Laboratory of Precision Chemistry and Molecular Engineering, Shanghai Key Laboratory of Functional Materials Chemistry, Feringa Nobel Prize Scientist Joint Research Center, Institute of Fine Chemicals, Frontiers Science Center for Materiobiology and Dynamic Chemistry, School of Chemistry and Molecular Engineering, East China University of Science and Technology, Shanghai 200237, China.

[3]State Key Laboratory of Green Chemical Engineering and Industrial Catalysis, Center of Photosensitive Chemicals Engineering, East China University of Science and Technology, Shanghai, 200237, China.

[4]Shanghai Frontier Science Research Base of Optogenetic Techniques for Cell Metabolism, School of Pharmacy, East China University of Science and Technology, Shanghai, 200237, China.

***Corresponding author**. guozq@ecust.edu.cn; yyuan@ecust.edu.cn; liucs@ecust.edu.cn

Contents

**1.**	**Experimental section**	**S3-S7**
**2**	**Synthesis of NIR-II quinolinium cyanine dyes QC7CN**	**S8-S11**
**3.**	**Summary of recently reported photothermal agents**	**S12**
**4.**	**Thermogravimetry analysis of QC7 dye**	**S13**
**5.**	**Characterization of QC7CN encapsulated within DSPE-mPEG2000**	**S13**
**6.**	**Characterization of ultrafine calcium phosphate oligomers CPO**	**S13**
**7.**	**Comparation between of GelMA-CPO scaffold and GelMA scaffold**	**S14**
**8.**	**Photothermal penetration depth comparison of different scaffolds**	**S15**
**9.**	**Characterization of thermosensitive copolymer (PNAm) and microsphere (MS)**	**S16**
**10.**	**Photothermal-triggered PTH release of QC7CN+PMS@GC scaffolds at different PMS loading**	**S17**
**11.**	**PTH stability after photothermal treatment**	**S18**
**12.**	**Biocompatibility of photothermal-responsive scaffolds**	**S18**
**13.**	**Photo-triggered pulsatile PTH release for osteogenic differentiation**	**S19**
**14.**	**PCR primer sequences**	**S20**
**15.**	**Alizarin red S (ARS) staining about osteogenic differentiation**	**S20**
**16.**	**Deep-tissue pulsatile PTH delivery for osteoporotic bone repair**	**S21**

1. Experimental section

Preparation of photothermal-responsive agents (PTA)

ICG (1 mg) was dissolved in 1 ml Ultrapure water, and the blue transparent solution was obtained by ultrasonic dispersion. It was subsequently diluted to 100 μg mL^{-1} using ultrapure water, as required.

QC7CN or QC7 (1mg) and DSPE-mPEG2000 (30mg) were mixed in a centrifuge tube and dissolved in DMSO (1mL). The solution was then slowly dripped into 9 ml of ultrapure water under ultrasound. The resulting mixed solution was dialysed in ultrapure water for 24 h and DMSO was removed to give an aqueous solution of QC7CN or QC7 micelles of 100 μg mL^{-1}. The concentration can be adjusted by ultracentrifugation according to the needs of the experiment.

Evaluation of photothermal-responsiveness performance

In order to test the photothermal conversion properties of QC7CN micelles, 0.5 mL of QC7CN micelles aqueous solution was placed in a 2 mL centrifuge tube and irradiated with a laser at 1064 nm with a laser intensity of 1.0 W cm^{-2} (the maximum permissible exposure (MPE) of 1064 nm). Real-time temperature and in-situ thermal images of aqueous solutions of QC7CN micelles at different concentrations (0, 50, 100, 200 ug mL^{-1}) were monitored using a thermal imaging system. The evaluation method for the photothermal conversion performance of IGC and QC7 is consistent with the above.

In order to compare the photothermal conversion capabilities of ICG, QC7 and QC7CN in deep tissues, they were irradiated with infrared light at 808 nm, 980 nm and 1064 nm penetrating gradually thickened chicken breast tissues (0, 2, 4, 6, 8, 10, 12, 14 mm), respectively. The real-time temperature of PTA aqueous solution was monitored using a thermal imaging system. The experiment concluded once the temperature increase of the solution reached 4 °C, determining the maximum penetration depth achievable in the current thickness of the chicken breast tissue.

Calculation of photothermal conversion efficiency

QC7CN micelles aqueous solution (100 μg mL^{-1}) reached the maximum temperature plateau after 10min irradiation with 1064 nm laser (1.0 W cm^{-2}), then the laser was turned off and the temperature curve of natural cooling to room temperature was recorded. From this, the photothermal conversion efficiency of QC7CN was calculated to be 49.3%. The calculation process is roughly as follows:

$$\eta = \frac{hS(T_{Max} - T_{Surr}) - Q_{Dis}}{I(1 - 10^{-A1064})} \text{......(1)}$$

where h is the heat transfer efficiency ; S is the surface area of the container (0.785 cm^2); T_{Max} and T_{Surr} are the maximum plateau temperature (66 °C) and ambient temperature (24 °C), respectively; Q_{Dis} is the heat dissipation due to light absorption by the solvent and container, Q_{Dis} can be calculated from equation (4). I is the intensity of the irradiated laser (1.0 W cm^{-2}), and A_{1064} is the absorbance of the sample at a wavelength of 1064 nm. hS can be calculated from equation (2):

$$\tau_s = \frac{m_D C_D}{hS} \text{......(2)}$$

Here m_D and C_D represent the mass of the solution (0.5 g) and the specific heat capacity of the solvent (pure water) (4.2 J g^{-1}), respectively. τ_s can be calculated from equation (3):

$$t = -\tau_s \ln\left(\frac{T_{RT} - T_{Surr}}{T_{Max} - T_{Surr}}\right) \text{......(3)}$$

Here T_{RT} represents the real-time temperature during the cooling process, and τ_s is calculated to be 317.46 s.

$$QDis = \frac{m_D c_D (T_{Max(water)} - T_{Surr})}{\tau_{s(water)}} \ldots\ldots(4)$$

According to the obtained data and formula (1), the photothermal conversion efficiency of QC7CN was calculated to be 49.3%.

Preparation and characterization of PTH drug-loaded thermosensitive microsphere (PMS)

Preparation of PMS: *N*-acryloyl glycinamide (NAGA) 120 mg, acrylamide (AAM) 55mg and appropriate photoinitiator LAP were dissolved in 0.6 mL ultrapure water and appropriate PTH (1-34) (teriparatide) was added as required. The solution was added to a 12-well plate and solidified into temperature-sensitive hydrogel (PNAm) after irradiation with blue light at 405 nm for 2h. PNA was heated to 45 ^{o}C and then incubated for 10 min to make it completely sol. Using Span80 as emulsifier, the solified PNAm was added into mineral oil slowly and stirred for several hours. After cooling, the PTH-loaded temperature-sensitive microspheres (PMS) were obtained. Then, ethanol was used as demulsifier to wash away mineral oil and Span80, and PMS was obtained by natural volatilization to remove ethanol. The product was stored at -20 ^{o}C.

Characterization of PMS: Rheological tests were performed on a rotational rheometer (Thermo Hakke) with parallel plates of 10 mm radius to evaluate the phase transition temperature of PMS. 0.2 g PMS was added to a parallel plate with a radius of 10mm and the test temperature was adjusted from 27 ^{o}C to 57 ^{o}C. The storage modulus (G') and loss modulus (G") of the microspheres were measured after 5 min incubation at each temperature using a stepwise heating method (3 ^{o}C) to determine their current state. The morphology of PMS was evaluated using light microscopy and images were analyzed by ImageJ software to generate particle size histograms. Calcein was loaded into MS to form Ca-MS to simulate PTH in MS. Meanwhile, Ca-MS was heated to 41 ^{o}C and incubated for 3 min, and its morphology was observed again by optical microscope to simulate release behavior by Ca-MS thermal degradation.

Preparation and characterization of photothermal-responsive scaffolds

Preparation of QC7CN@GC: The main printing matrix of the 3D printing scaffold are methyl acrylate gelatin (GelMA) and calcium phosphate oligomer (CPO). The photothermal response function is achieved by loading photothermal-responsive agents into 3D printing scaffold GelMA-CPO (GC). Taking QC7CN@GC as an example, the specific procedure is as follows: first, prepare 2 mL of GelMA-CPO 3D-printed matrix according to the aforementioned method. Then, add concentrated QC7CN micelles to prepare a slurry with a concentration of 100 µg mL^{-1}. The slurry was filled into temperature-controlled photocuring barrel, and printed into the pre-set shape after temperature stabilized at 18 ^{o}C.

Preparation of QC7CN+PMS@GC: After the dried PMS were dissolved in water for 5 minutes, excess water was filtered out through a φ = 40 µm filter screen. Then, add an appropriate proportion of 3D printing raw materials, mix evenly. The slurry was filled into a temperature-controlled photocuring barrel, and printed into the pre-set shape after the temperature stabilized at 18 ^{o}C.

Characterization of GC, ICG@GC, QC7@GC, QC7CN@GC: Mechanical test: G and GC were prepared into φ = 8 mm, h = 5 mm circular stents using 3D printer, and mechanical compression test was carried out by electronic universal testing machine (CMT6103) to evaluate the change of mechanical properties. FTIR: GC and ICG/QC7/QC7CN@GC were tested using ATR-FTIR methods to determine the incorporation of CPO into GelMA to form composite scaffolds and ICG and QC7CN into GC to form photothermal responsive 3D printable scaffolds. Degradation: 0.5 g PTA@GC was placed in 30 ml of PBS in a shaker at 37 ^{o}C for 14 days. Samples were taken on the first day, lyophilized, weighed and recorded. Thereafter, records were taken every two days with 3 parallel samples. Photothermal properties: ICG@GC, QC7@GC and QC7CN@GC were placed in a plastic dish and irradiated with 80 8nm, 980 nm and 1064 nm at their respective MPE intensities. Real-time temperature and in-situ thermal images of the three were monitored using thermal imaging systems. At the same time, chicken breast was used as an obstacle to test the photothermal response capabilities of the three in deep tissues. Specific test methods

refer to the evaluation of photothermal conversion ability in deep tissues.

Characterization of PMS@GC: Morphology observation: PMS and GC were freeze-dried, and the microstructure of PMS and PMS@GC with different PMS content was observed by field emission scanning electron microscope (SEM). Mechanical testing: Mechanical compression testing was performed using an electronic universal testing machine (CMT6103) to evaluate the mechanical strength of PMS@GC with different PMS content.

Photothermal-triggered PTH release

Using 6 mm thick chicken breast as a barrier, different amounts of ICG+PMS@GC and QC7CN+PMS@GC were placed in 12-well plates and 4 mL PBS was added. Irradiate ICG+PMS@GC and QC7CN+PMS@GC with MPE light intensity of 808 nm and 1064 nm respectively for 1 min and then stop. After standing for 20 min, take 500 μL and supplement with PBS. The plate was then placed on a constant temperature shaker at 37 °C. After 24 h, 500 μL was sampled again and supplemented with 500 μL PBS. Repeat this process 6 times and collect PTH release at each step. The PTH concentration in each sample was analyzed by ELISA kit. After calculation, the PTH content released by nature and photothermal in different stents was obtained.

Cell experiment

Adhesion and proliferation of BMSCs

Isolation of bone marrow macrophages (BMMs): Since osteoclasts are derived from bone marrow macrophages through induced culture, it is necessary to first isolate bone marrow macrophages from SD rats. First, 8-week-old male SD rats were euthanised and immersed in 75% ethanol for 5 min for sterilisation. The rats were then transferred to a laminar flow hood, and the bilateral femurs and tibias were removed; the cartilaginous caps at both ends of the bones were trimmed off. Subsequently, the bone marrow cavities were flushed with 10 mL of complete medium C (αMEM supplemented with 10% foetal bovine serum and 2% penicillin-streptomycin) to collect the cells. The collected cells were centrifuged at 1400 rpm at room temperature for 5 min, the supernatant was discarded, and the cells were resuspended in 10 mL of complete medium C. Transfer the resuspended cells to a cell culture dish and incubate overnight in a cell culture incubator (37 °C, 5% CO_2). After overnight culture, collect the supernatant and centrifuge it at 1400 rpm at room temperature for 5 min. Discard the supernatant, add 3 mL of erythrocyte lysis buffer to the centrifuge tube, mix thoroughly by pipette pipetting, and wait for 90 seconds to ensure that the erythrocytes are completely removed. Subsequently, add 10 mL of PBS to stop the reaction, and centrifuge again at 1400 rpm at room temperature for 5 min to collect the cells. Resuspend the collected cells in 10 mL of complete medium C, and determine the cell count using a hemocytometer.

Induction of osteoclasts (OCs): Cell seeding was performed using a 24-well plate as an example. The counted cells were diluted to a concentration of 3×10^5 cells/mL, and 1 mL cell suspension was added to each well of 24-well plate. Subsequently, M-CSF was added to each well, ensuring a final concentration of 60 ng/mL. The 24-well plate is then placed in cell culture incubator (37 °C, 5% CO_2) for 24 h. After 24 hours, when the BMMs have almost completely adhered to the surface, RANKL is added to induce the fusion of BMMs into osteoclasts (OCs), maintaining a final RANKL concentration of 40 ng/mL. Following 72 hours of induction, usable OCs are obtained for the next stage of the experiment.

In vitro cytotoxicity assay

Different scaffolds' extract was prepared at a concentration of 0.2 g mL^{-1}. BMSCs were seeded in 96-well plates at a density of 5×10^3 cells per well, and the prepared extract was added to co-culture with BMSCs after the cells adhered to the wall. MTT assay was used to evaluate

the biocompatibility of different PTA at 1, 4, and 7 days.

Osteogenic gene expression in BMSCs

BMSCs were seeded in 6-well plates at a density of 1×10^5 cells per well, and different materials were added after cells adhered to the wall. The groups were as follows: (1) blank group (no material), (2) GC group, (3) PMS@GC (PTH sustained release group), (4) GC with factitious PTH injection (PTH (10 nM) was added daily instead of PTH delivery device), (5) ICG+PMS@GC (808 nm laser irradiation group) and (6) QC7CN+PMS@GC (1064 nm laser irradiation group). The laser irradiation power was MPE intensity, and the irradiation time was 1 min for 4 days. After laser irradiation, 6 mm chicken breast was covered on the perforated plate to distinguish the photothermal penetration of different PTA. Osteogenic induction medium (Oricell, RAXMX-90021) was used to replace the original medium during culture. After four days of culture in the above manner, BMSCs were lysed by using TRIZOL (TAKARA, Japan) and purified to obtain a total RNA extract. Purity and concentration were quantified using NanoDrop 2000 (Invitrogen, USA). Reverse transcription was performed using PrimeScript RT kit (TAKARA,RR 037A). PCR was performed using TB Green Premix Ex Taq kit (TAKARA,RR 420A) according to manufacturer's instructions. GAPDH was used as a reference gene.

Osteogenic differentiation of BMSCs

BMSCs were seeded into 24-well plates, 2×10^4 cells per well. After cells adhered to the wall, different materials were added according to the above groups. The laser irradiation method and culture method are the same as above, and the culture time is extended to 7 days. After 7 days of culture, the osteogenic differentiation of the different groups was further assessed using alkaline phosphatase (ALP) assay. For the ALP activity test, 100 μL of p-nitrophenyl phosphate solution (2.5 mg mL^{-1}) was incubated with 50 μL of lysate until staining. In addition, osteogenic potential was tested using BCIP/NBT alkaline phosphatase chromogenic kit (Beyotime, Shanghai). In addition, alizarin red staining (ARS) was used to characterize osteogenic differentiation. Cells were cultured as described above and the culture time was extended to 21 days. Cells were fixed with 4 % paraformaldehyde. Fixed cells were then washed twice with PBS and stained with 1 % alizarin red (pH = 4.2) for 30 minutes. Then, the residual alizarin red dye was washed with PBS and images were taken.

Animals

Modeling and repair of osteoporotic bone defect

First, 10-month-old female SD rats underwent bilateral ovariectomy (OVX) after a 3-day acclimation period, and were kept for 6 weeks to establish osteoporosis models. A cylindrical defect of φ = 3mm, h = 2 mm was made on both femoral condyles of each osteoporosis rat, and the repair material was filled into the defect, followed by suture of the wound. They are grouped as follows: (1) blank group (no filler material), (2) GC group, (3) PMS@GC (PTH sustained release group), (4) GC with factitious PTH injection (PTH (40μg/kg/d) was subcutaneously injected for 14 days without PTH delivery device), (5) ICG+PMS@GC (808 nm laser irradiation group) and (6) QC7CN+PMS@GC (1064 nm laser irradiation group). The laser irradiation power was MPE intensity, and the irradiation total time was 1min for 7 days. Ethical approval for the animal experiments was obtained prior to the research from East China University of Science and Technology Animal Studies Committee, and the assigned approval number was ECUST-2025-200.

Micro-CT analysis of bone repair

After 4 and 8 weeks of repair, all rats were sacrificed and femurs removed and fixed with 4 % paraformaldehyde. To determine the formation of new bone, samples were scanned using a Micro-CT system and 3D images reconstructed. In addition, the parameters of bone defect site were evaluated by analysis software, including bone mineral density (BMD), bone volume percentage (BV/TV), trabecular number (Tb.N),

trabecular spacing (Tb.Sp), etc.

Histological and Immunohistochemical Analysis

To assess bone histology, samples were fixed in 4% paraformaldehyde for 48 hours, then decalcified in 10 % EDTA for 6 weeks, dehydrated with graded alcohol and embedded in paraffin, and serial sections 5μm thick were cut for HE, Masson, IHC and TRAP staining analysis. For IHC staining, slides were processed by heat-induced epitope retrieval using microwave heating in 0.01 M citrate buffer for 9 minutes. Sections were incubated in diluted normal serum for one hour and then incubated at 4 ^{o}C with primary antibodies, including anti-osteopontin (OPN) antibody (dilution 1:120, Abcam) and Runt-associated transcription factor 2 (Runx2) antibody (dilution 1:120, Abcam). After that, the sections were washed three times with PBS and incubated with biotinylated secondary antibody (dilution 1:150) for 1 hour.

2. Synthesis of NIR-II quinolinium cyanine dyes QC7CN

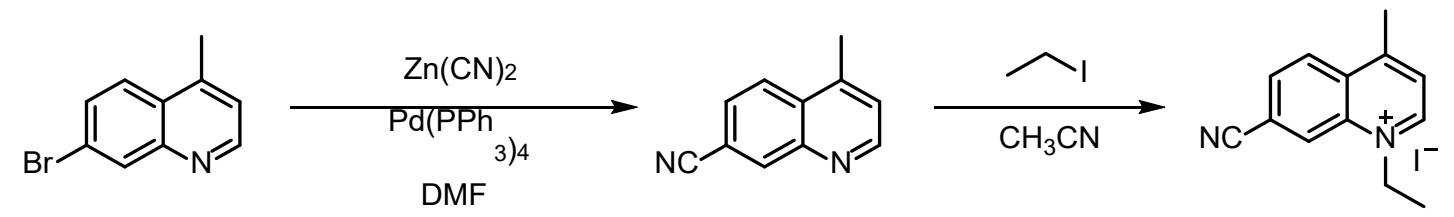

4-methylquinoline-7-carbonitrile: 7-bromo-4-methylquinoline (200 mg, 0.90 mmol, 1 equiv), $Zn(CN)_2$ (211 mg, 1.80 mmol), and $Pd(PPh_3)_4$ (93 mg, .0.09 mmol, 0.2 equiv) were dissolved in DMF (5 mL) in a 50 mL Schlenk Tube and heated to 95 °C for 24 h under an argon atmosphere. The solution was cooled to rt, quenched with the addition of 10 mL aq. $NH_4Cl/NH_3{\bullet}H_2O$ (v/v, 4/1), filtered through a pad of silica gel, extracted three times with EtOAc, dried with Na_2SO_4, filtered. The resulted filtrate was concentrated under reduced pressure, and then the crude product was purified by silica gel chromatography using PE/DCM (v/v, 75/25) as the eluent to afford 4-methylquinoline-7-carbonitrile as a white solid (145 mg, 0.86 mmol): Yield 95%.**All glassware were cleaned by aq. NH_4OH and NaClO**. ^{1}H-NMR (400 MHz, $CDCl_3$, ppm): δ 2.75 (s, 3H, CH_3-Ph), 7.39 (d, J = 4.4 Hz, 1H, Ph-H), 7.73 (d-d, J_1 = 8.6 Hz, J_2 = 1.6 Hz, 1H, Ph-H), 8.11 (d, J = 8.8 Hz, 1H, Ph-H), 8.48 (d, J = 1.2 Hz, 1H, Ph-H), 8.90 (d, J = 4.0 Hz, 1H, Ph-H). ^{13}C-NMR (100 MHz, $CDCl_3$, ppm): δ 18.66, 112.59, 118.53, 124.18, 125.53, 126.99, 130.46, 135.89, 144.59, 146.91, 152.01. Mass spectrometry (ESI-MS, *m/z*): $[M + H]^+$ calcd. for $[C_{11}H_8N_2 + H]^+$ 169.0766; found: 169.0769.

7-cyano-1-ethyl-4-methylquinolinium: 4-methylquinoline-7-carbonitrile (145 mg, 0.86 mmol, 1 equiv) and iodoethane (2.68 g, 17.24 mmol, 20 equiv) were dissolved in dissolved in MeCN (2 mL) in a 50 mL Schlenk Tube and heated to 85 °C for 24 h under an argon atmosphere. The solvent was removed under reduced pressure, and then the crude product was purified by silica gel chromatography using DCM/MeOH (v/v, 90/10) as the eluent to afford 7-cyano-1-ethyl-4-methylquinolinium as a yellow solid (230 mg, 0.71 mmol): Yield 53%. ^{1}H-NMR (400 MHz, CD_3OD, ppm): δ 1.75 (t, J = 7.2 Hz, 3H, $-CH_3$), 3.11 (s, 3H, CH_3-Ph), 5.14 (q, J = 7.2 Hz, 2H, $-CH_2-N^+$), 8.15 (d, J = 6.0 Hz, 1H, Ph-H), 8.29 (d-d, J_1 = 8.8 Hz, J_2 = 1.2 Hz, 1H, Ph-H), 8.75 (d, J = 8.8 Hz, 1H, Ph-H), 9.17 (s, 1H, Ph-H), 9.45 (d, J = 6.0 Hz, 1H, Ph-H). ^{13}C-NMR (100 MHz, CD_3OD, ppm): δ 15.77, 20.50, 54.98, 118.01, 119.39, 126.01, 126.33, 130.14, 131.93, 132.75, 138.10, 151.08, 161.14. Mass spectrometry (ESI-MS, *m/z*): $[M]^+$ calcd. for $[C_{13}H_{13}N_2]^+$ 197.1079; found: 197.1071.

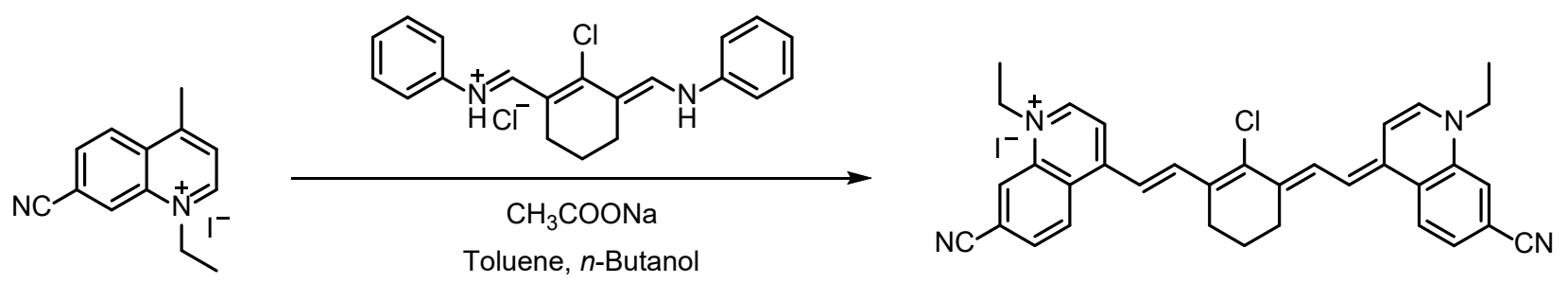

QC7CN

QC7CN: 7-cyano-1-ethyl-4-methylquinolinium (50 mg, 0.15 mmol, 1 equiv), *N*-[(3-(anilinomethylene)-2chloro-1-cyclohexen-1-yl)methylene]aniline hydrochloride (27 mg, 0.08 mmol, 0.5 equiv), and sodium acetate (19 mg, 0.23 mmol, 3 equiv) were added to a flame-dried 10 mL Schlenk Tube and dissolved in Toluene (1.0 mL) and *n*-Butanol (1.0 mL). The mixture was freeze-pump-thawed ×3 and heated to 100 °C for 12 h under an argon atmosphere. The reaction mixture was filtered and rinsed with additional DCM and H_2O to yield a black solid (37 mg, 0.056 mmol): Yield 73%. ^{1}H-NMR (400 MHz, DMSO-d_6, ppm): δ 1.39 (t, J = 6.8 Hz, 6H, $-CH_3$), 1.85 (t, 2H, $-CH_2-$), 2.81 (t, 4H, $-CH_2-$), 4.47 (q, J = 7.2 Hz, 4H, $-CH_2$-N), 7.07 (d, J = 13.6 Hz, 2H, Alkene-H), 7.45 (d, J = 7.6 Hz, 2H, Ph-H), 7.90 (d, J = 9.6 Hz, 2H, Ph-H), 7.98 (d, J = 13.6 Hz, 2H, Alkene-H), 8.16 (d, J = 7.2 Hz, 2H, Ph-H), 8.47 (s, 2H, Ph-H), 8.65 (d, J = 9.2 Hz, 2H, Ph-H). Mass spectrometry (ESI-MS, *m/z*): $[M]^+$ calcd. for $[C_{34}H_{30}N_4Cl]^+$ 529.2154; found: 529.5151. Absorbance (DCM): 552 nm, 923 nm, 1046 nm. Emission (DCM, ex. 885 nm): 1075 nm.

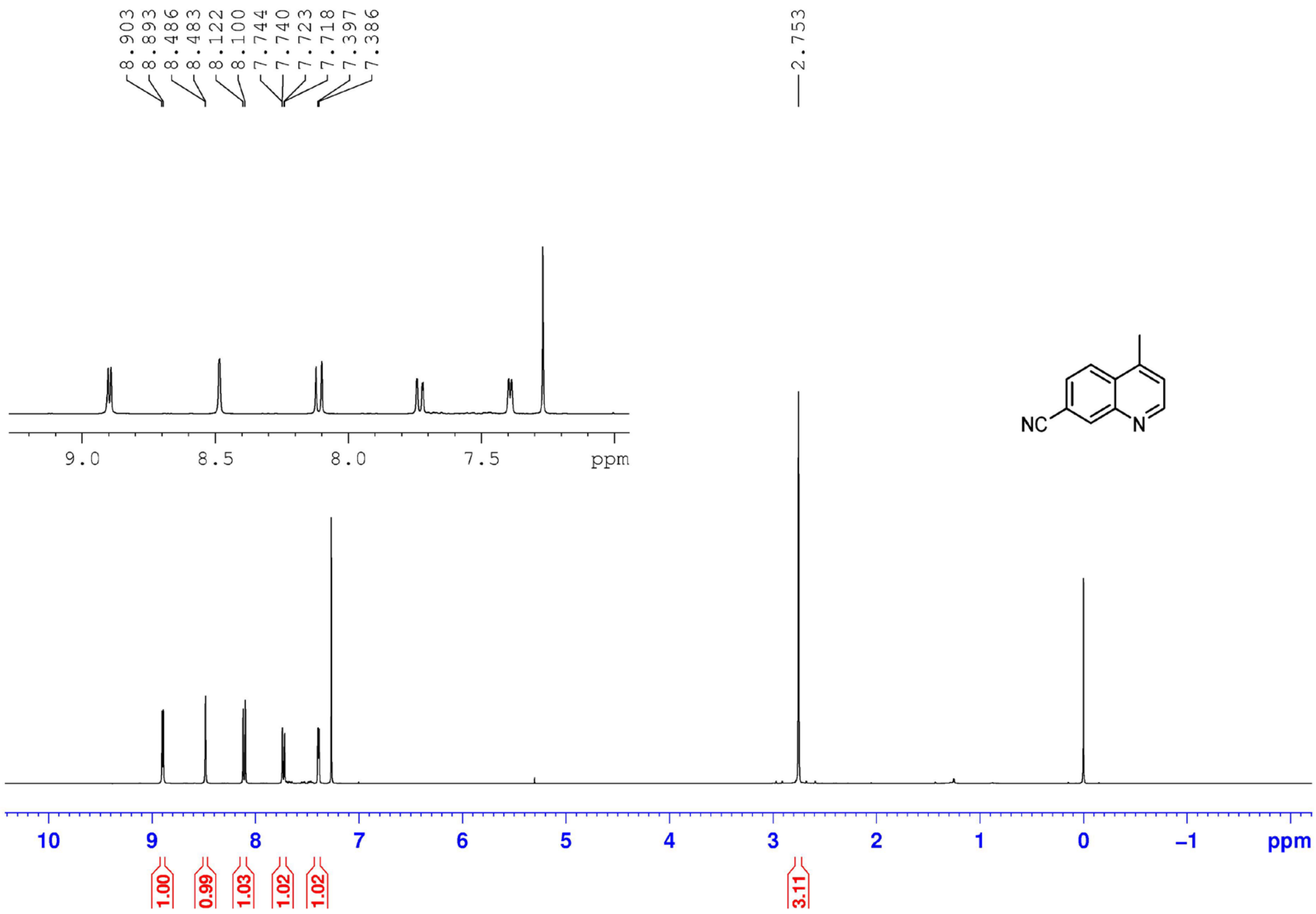

^{1}H NMR spectrum of 4-methylquinoline-7-carbonitrile in $CDCl_3$

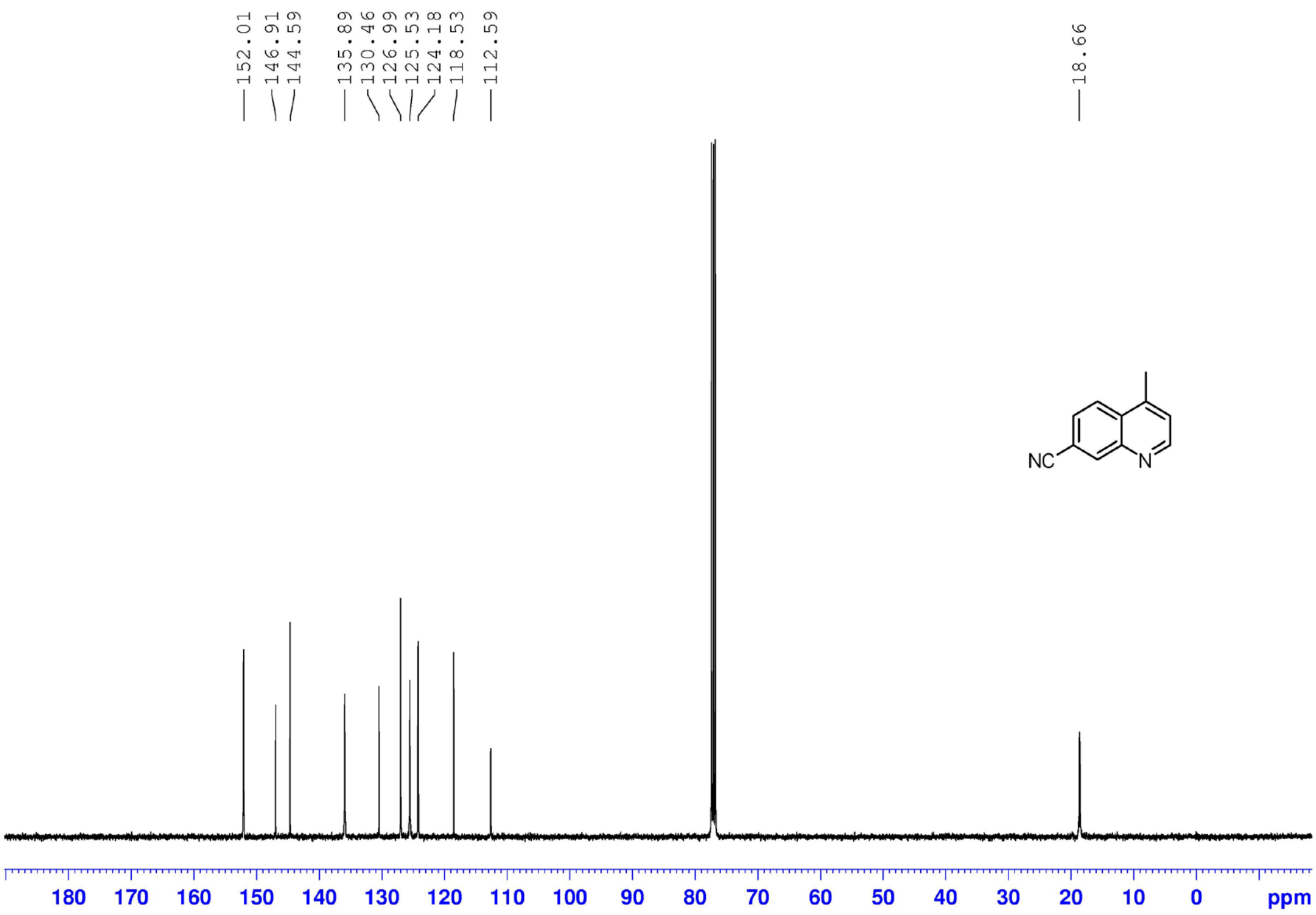

^{13}C NMR spectrum of 4-methylquinoline-7-carbonitrile in $CDCl_3$

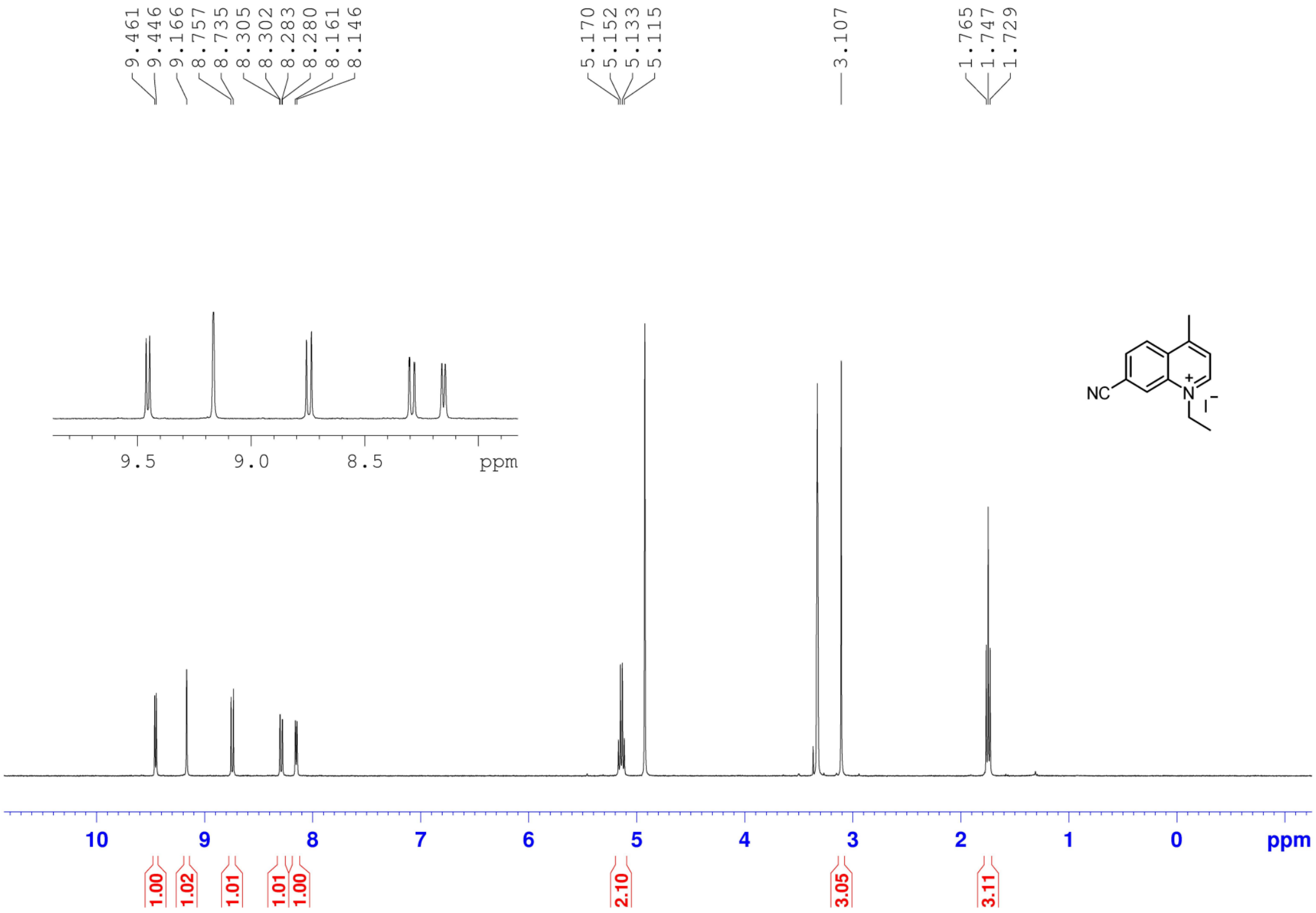

^{1}H NMR spectrum of 7-cyano-1-ethyl-4-methylquinolinium in CD_3OD

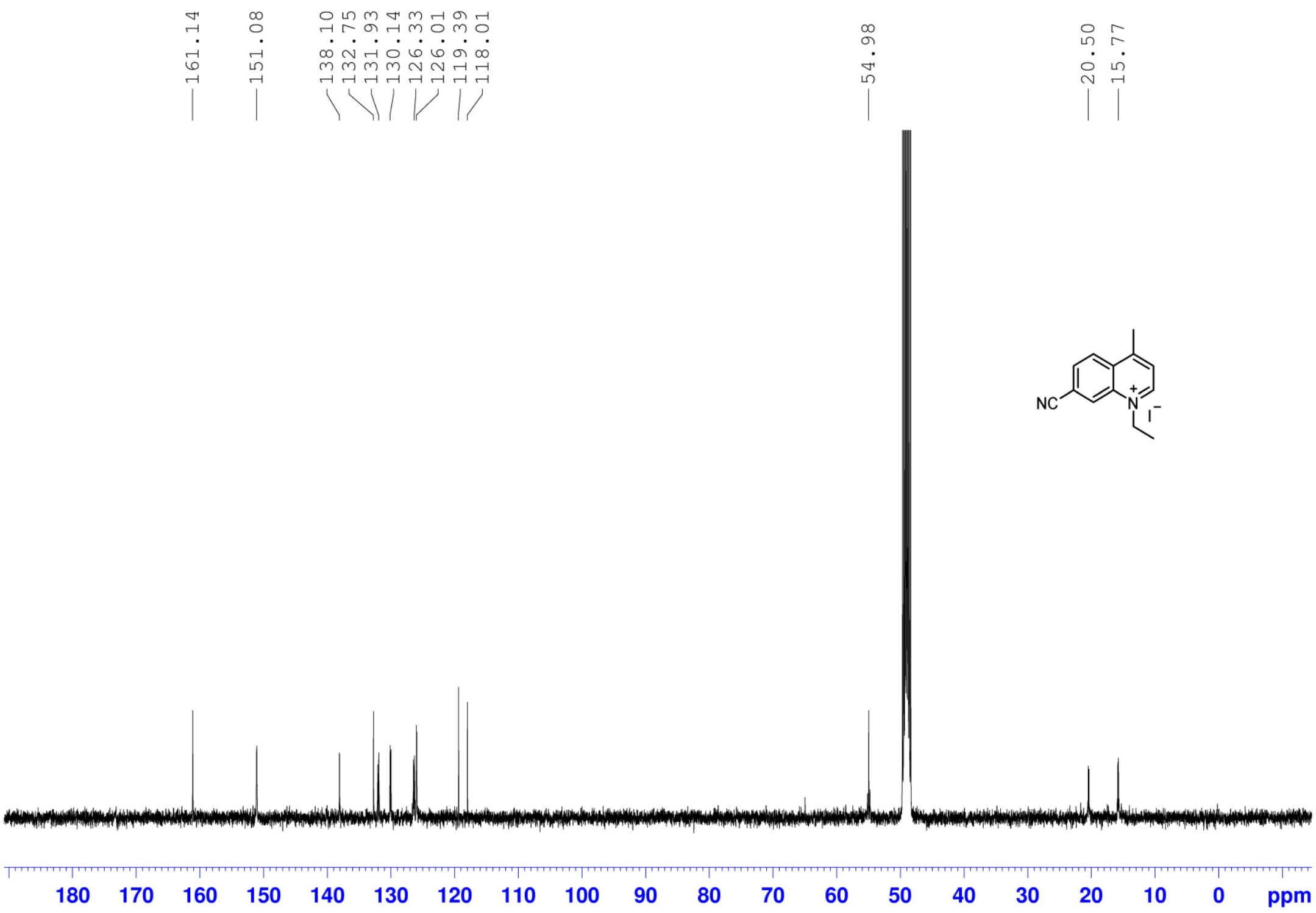

^{13}C NMR spectrum of 7-cyano-1-ethyl-4-methylquinolinium in CD_3OD

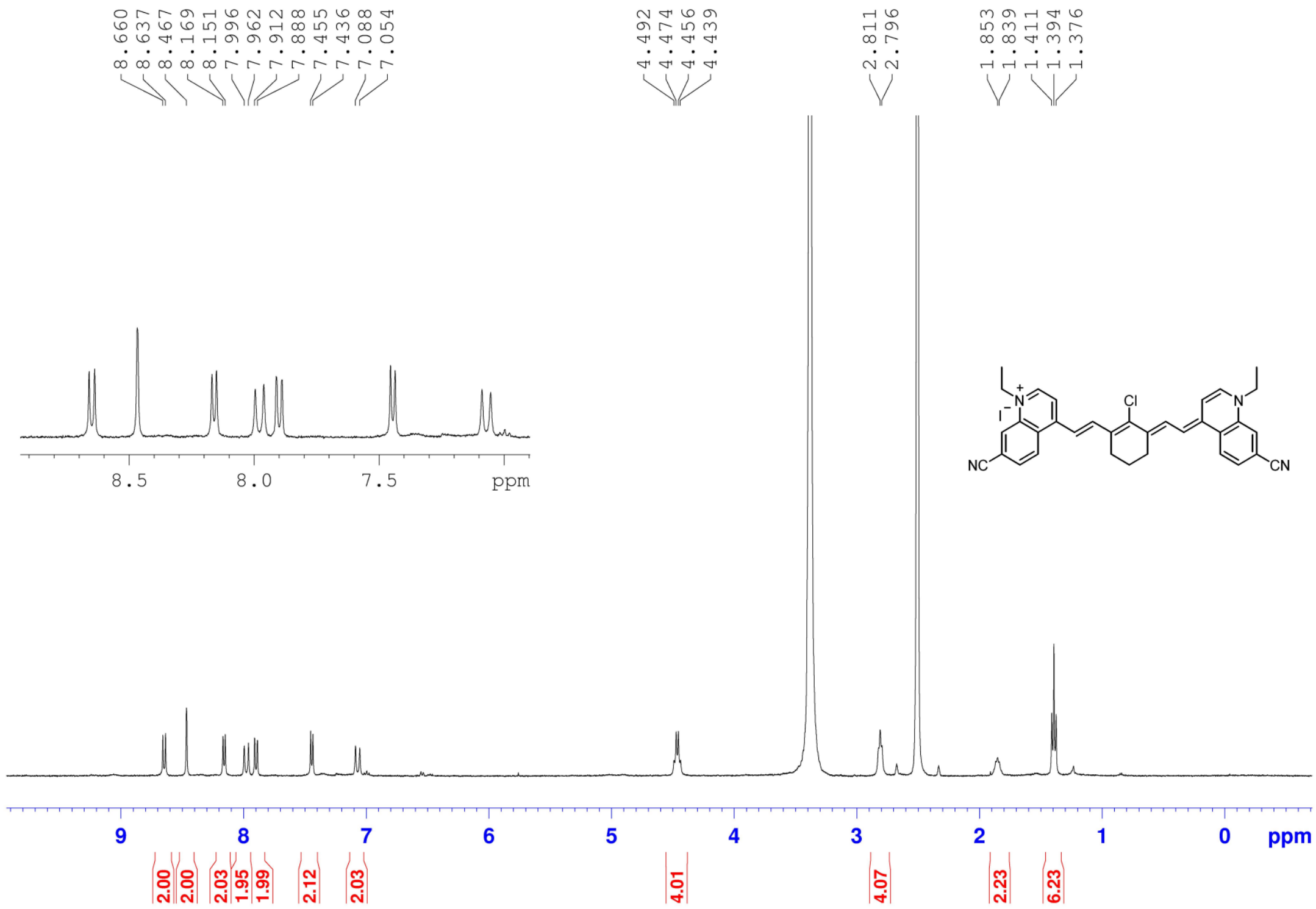

^{1}H NMR spectrum of QC7CN in DMSO-d_6

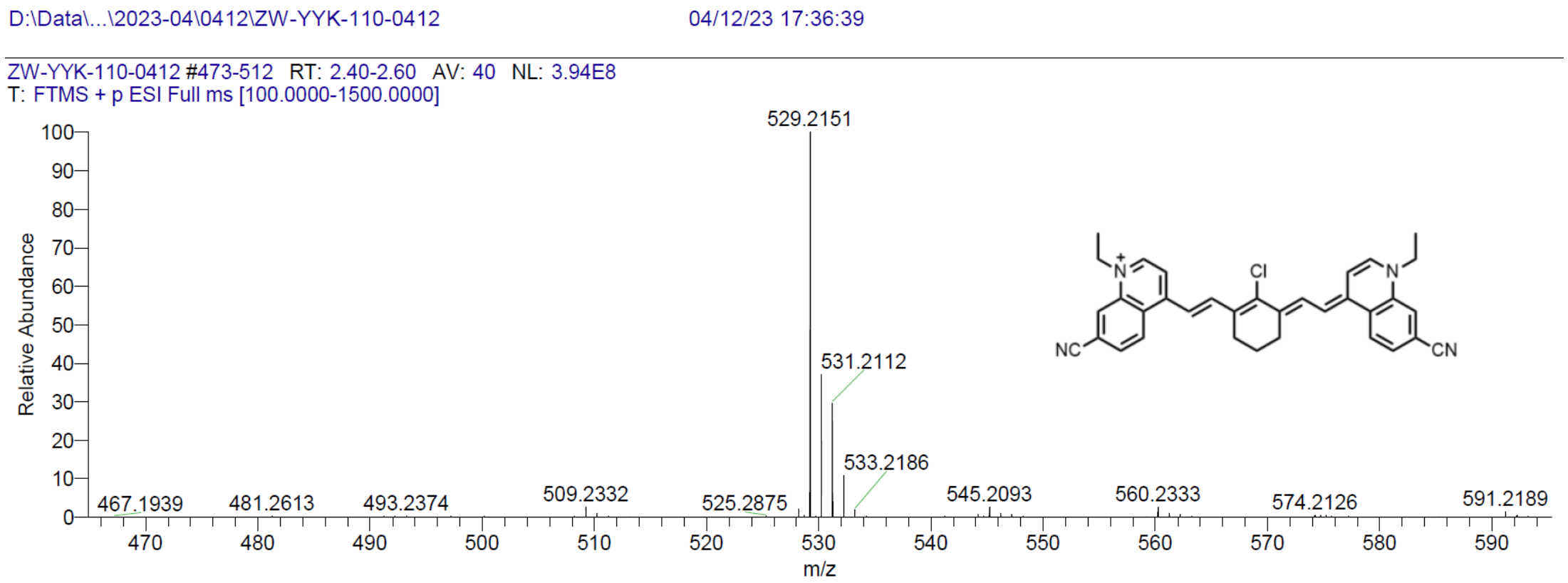

ZW-YYK-110-0412#473-513 RT: 2.40-2.60 AV: 41
T: FTMS + p ESI Full ms [100.0000-1500.0000]
m/z= 527.48-535.01

m/z	Intensity	Relative	Theo. Mass	Delta (ppm)	Composition
529.2151	396447328.0	100.00	529.2154	-0.26	$C_{34}H_{30}N_4Cl$

HRMS spectrum of compound QC7CN

Figure S1. Characterization of intermediate compounds and quinolinium dye.

3. Summary of recently reported photothermal agents

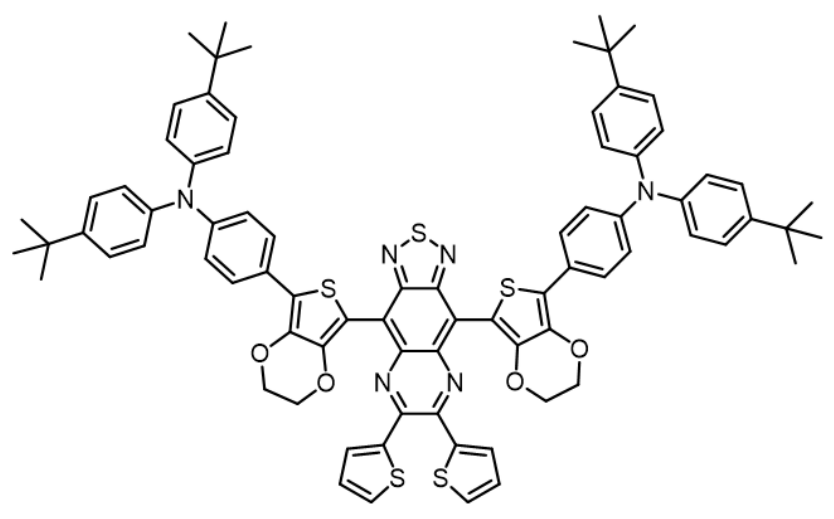

BTO-TTQ Molecular weight: 1343.8

$\lambda_{max,abs}$ = 774 nm, PCE = 48.9%

Adv. Mater. 2025, 37, 2502452

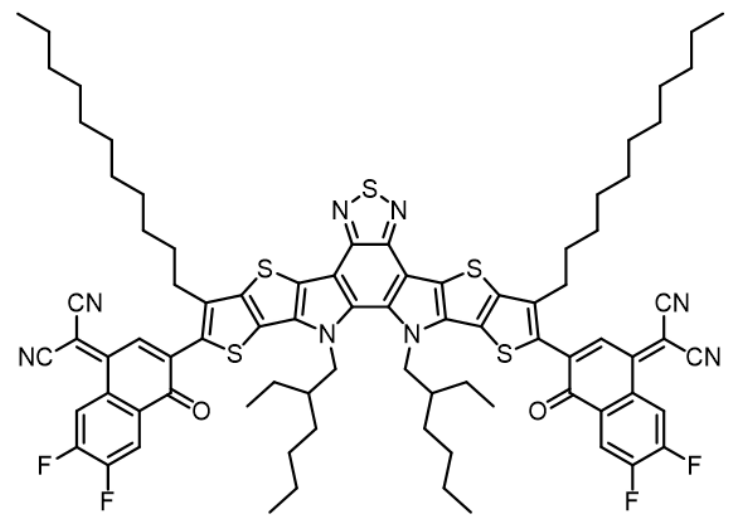

BPT-FNC Molecular weight: 1451.9

$\lambda_{max,abs}$ = 971 nm, PCE = 31.5%

Adv. Funct. Mater. 2025, 36, e17631

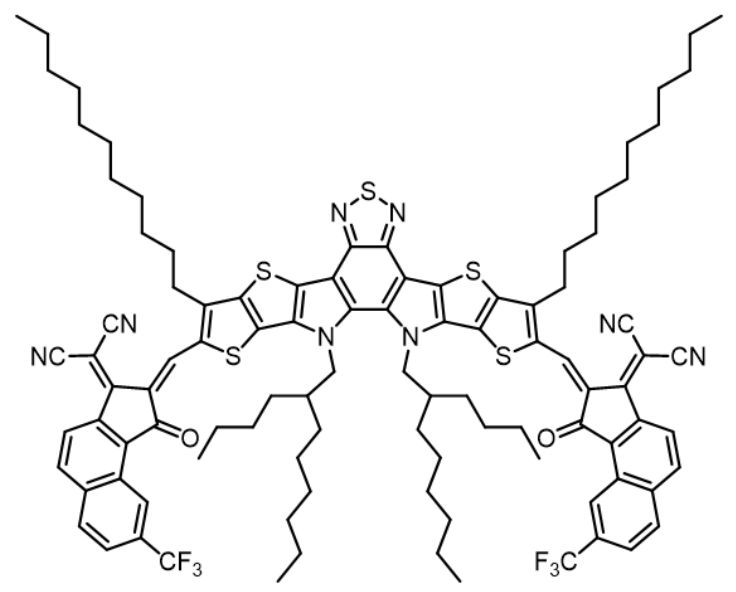

Y5-2BO-2BTF Molecular weight: 1728.3

$\lambda_{max,abs}$ = 789 nm, PCE = 72.8%

J. Am. Chem. Soc. 2025, 147, 3570-3583

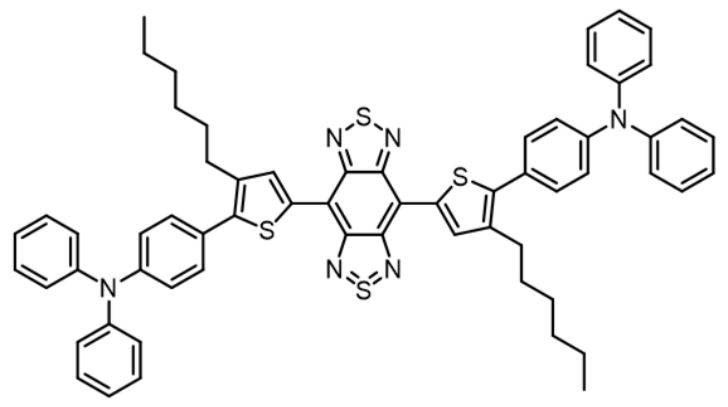

2TT-mC6B Molecular weight: 1013.4

$\lambda_{max,abs}$ = 810 nm, PCE = 32.4%

Adv. Mater. 2023, 36, 2307785

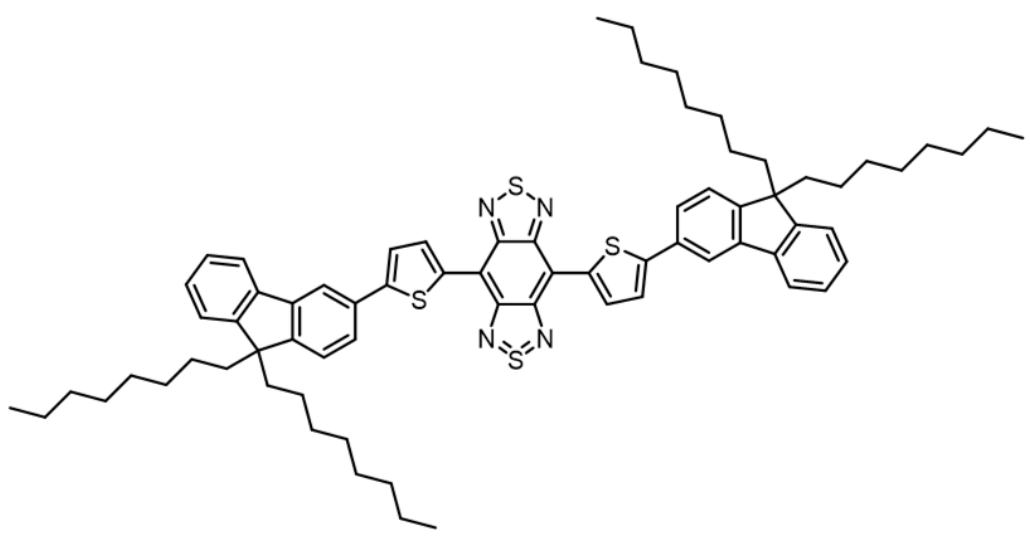

PM331 Molecular weight: 1135.7

$\lambda_{max,abs}$ = 813 nm, PCE = 40%

Proc. Natl. Acad. Sci. U.S.A. 2025, 24, e2503574122

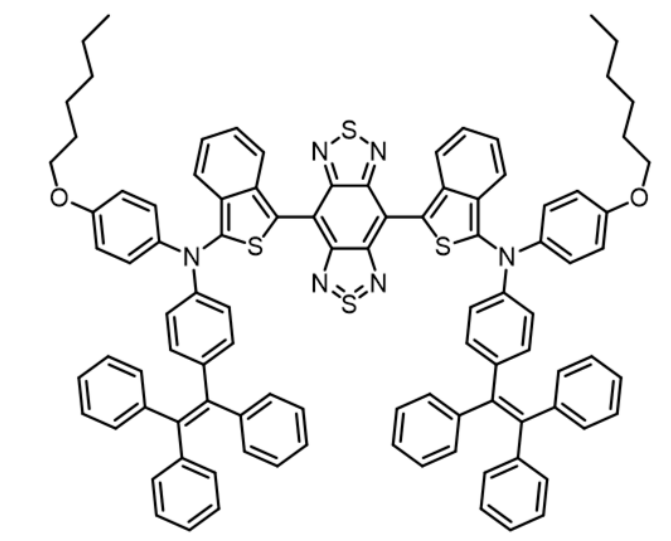

TPE-BT-BBTD Molecular weight: 1502

$\lambda_{max,abs}$ = 993 nm, PCE = 33.8%

Nat. Nanotechnol. 2024, 19, 834-845

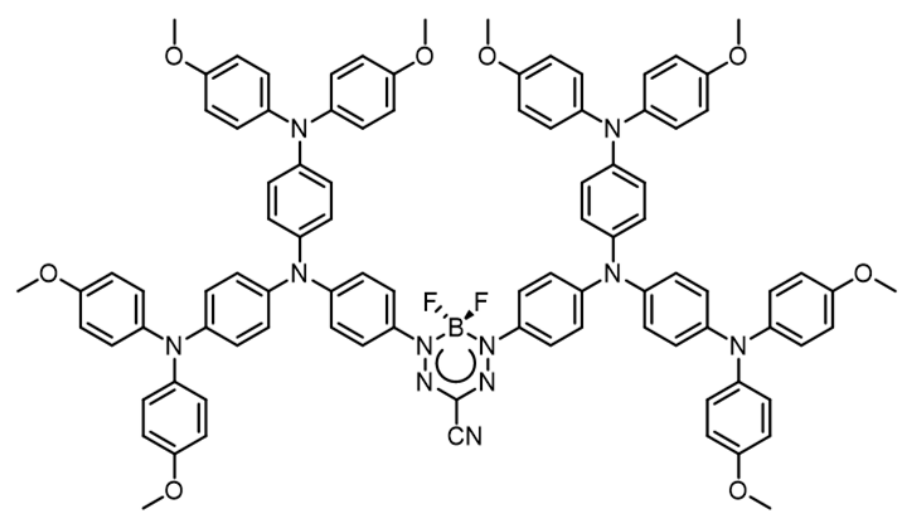

BDF-8OMe Molecular weight: 1541.6

$\lambda_{max,abs}$ = 905 nm, PCE = 62.5%

Angew. Chem. Int. Ed. 2025, 64, e202503718

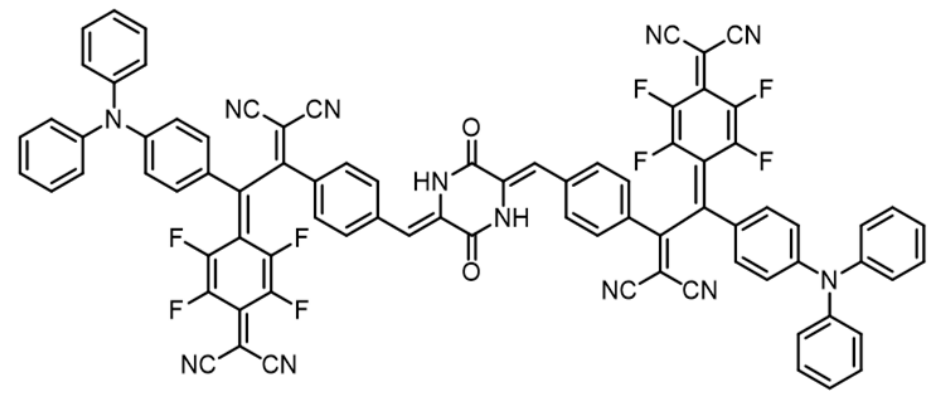

TPP Molecular weight: 1377.3

$\lambda_{max,abs}$ = 805 nm, PCE = 37%

ACS Mater. Lett. 2025, 7, 1060-1069

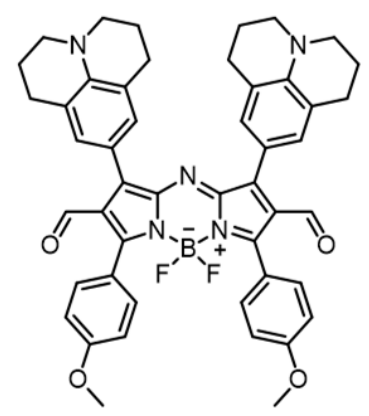

TTP Molecular weight: 803.7

$\lambda_{max,abs}$ = 1020 nm, PCE = 61%

Adv. Mater. 2023, 35, 2301901

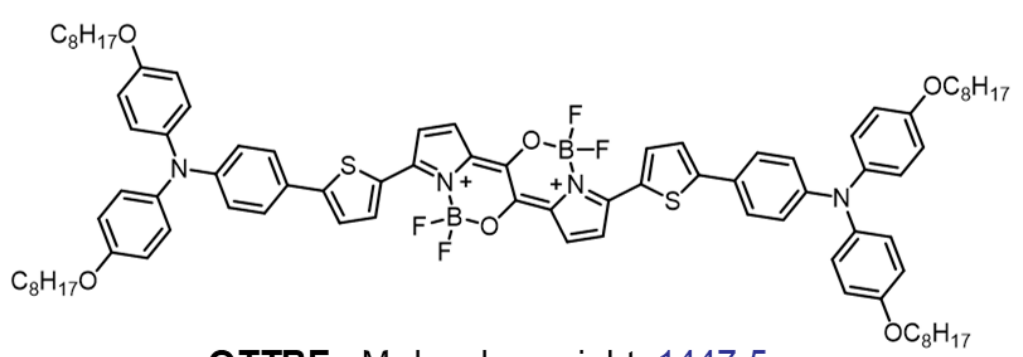

OTTBF Molecular weight: 1447.5

$\lambda_{max,abs}$ = 892 nm, PCE = 54.1%

Angew. Chem. Int. Ed. 2024, 63, e202407307

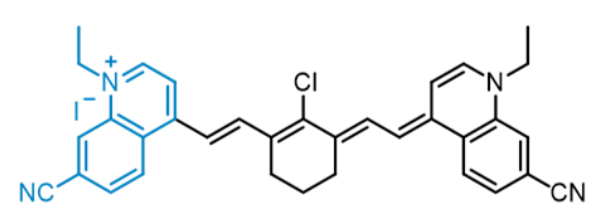

QC7CN Molecular weight: 657

$\lambda_{max,abs}$ = 1046 nm, PCE = 49.3%

This work, quinolinium dye

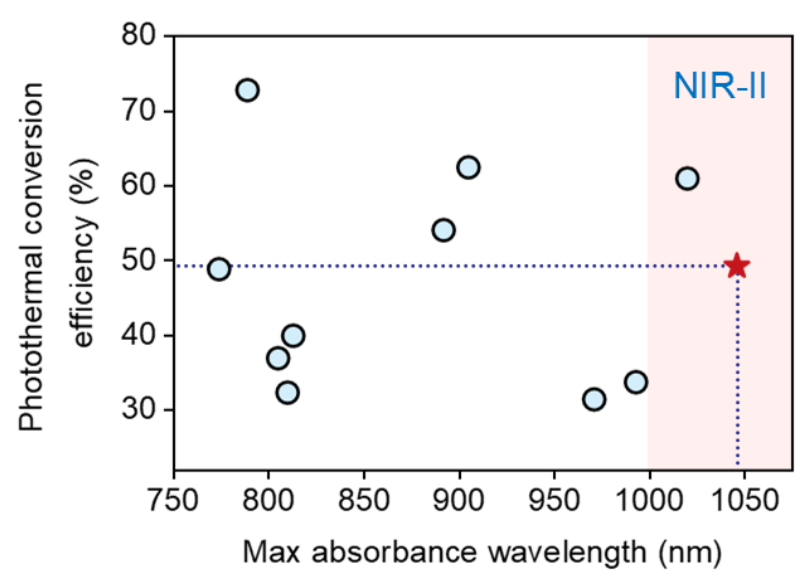

Figure S2. Comparison of the photothermal performance of reported organic photothermal agents.

4. Thermogravimetry analysis of QC7 dye

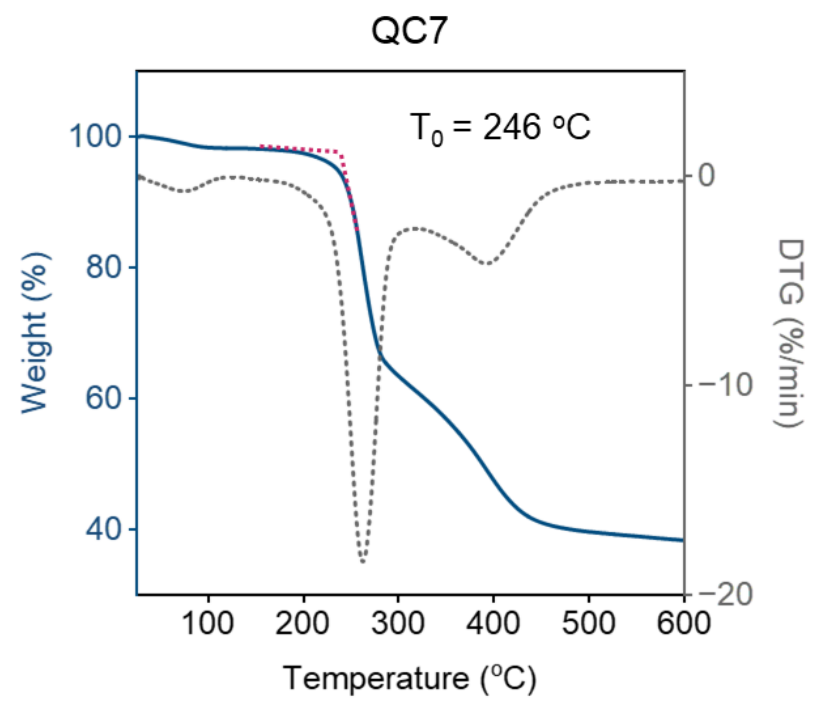

Figure S3. The Thermogravimetry analysis of QC7 dye.

5. Characterization of QC7CN encapsulated within DSPE-mPEG2000

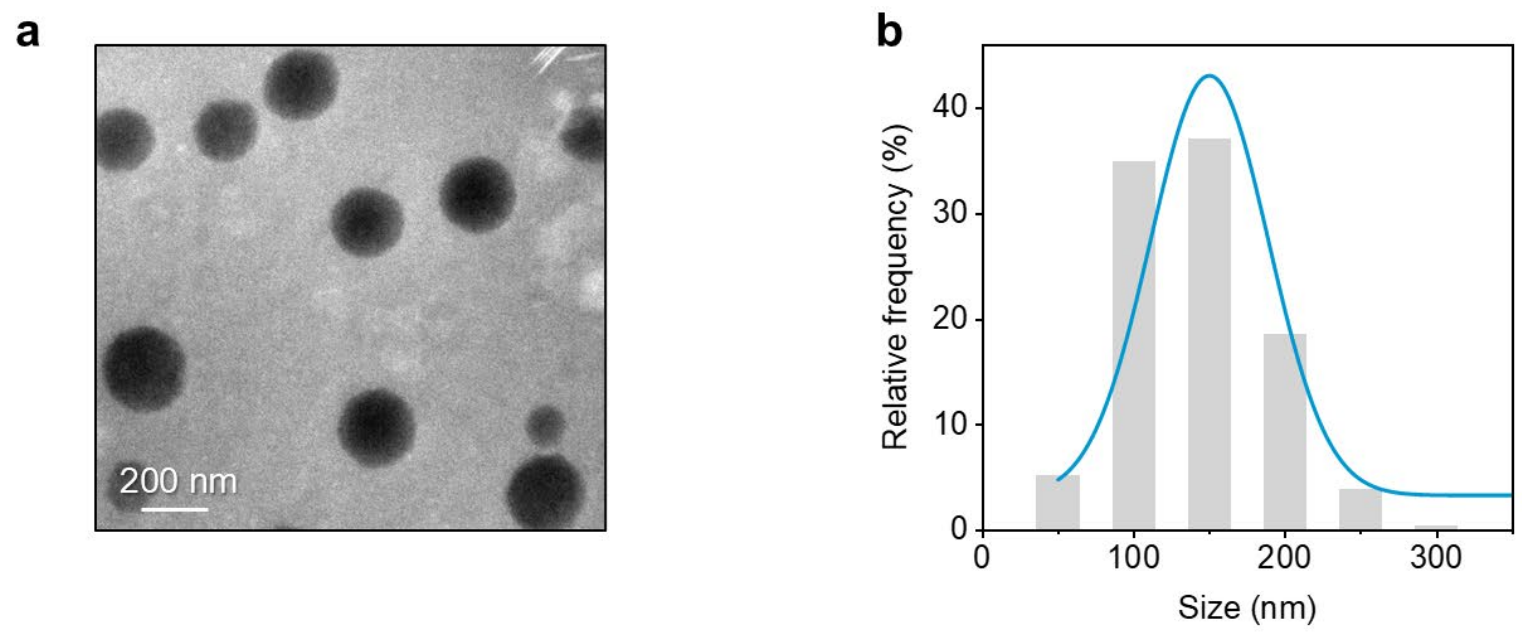

Figure S4. The transmission electron microscopy image (a) and size distribution (b) of QC7CN encapsulated within the DSPE-mPEG2000 polymer.

6. Characterization of ultrafine calcium phosphate oligomers CPO

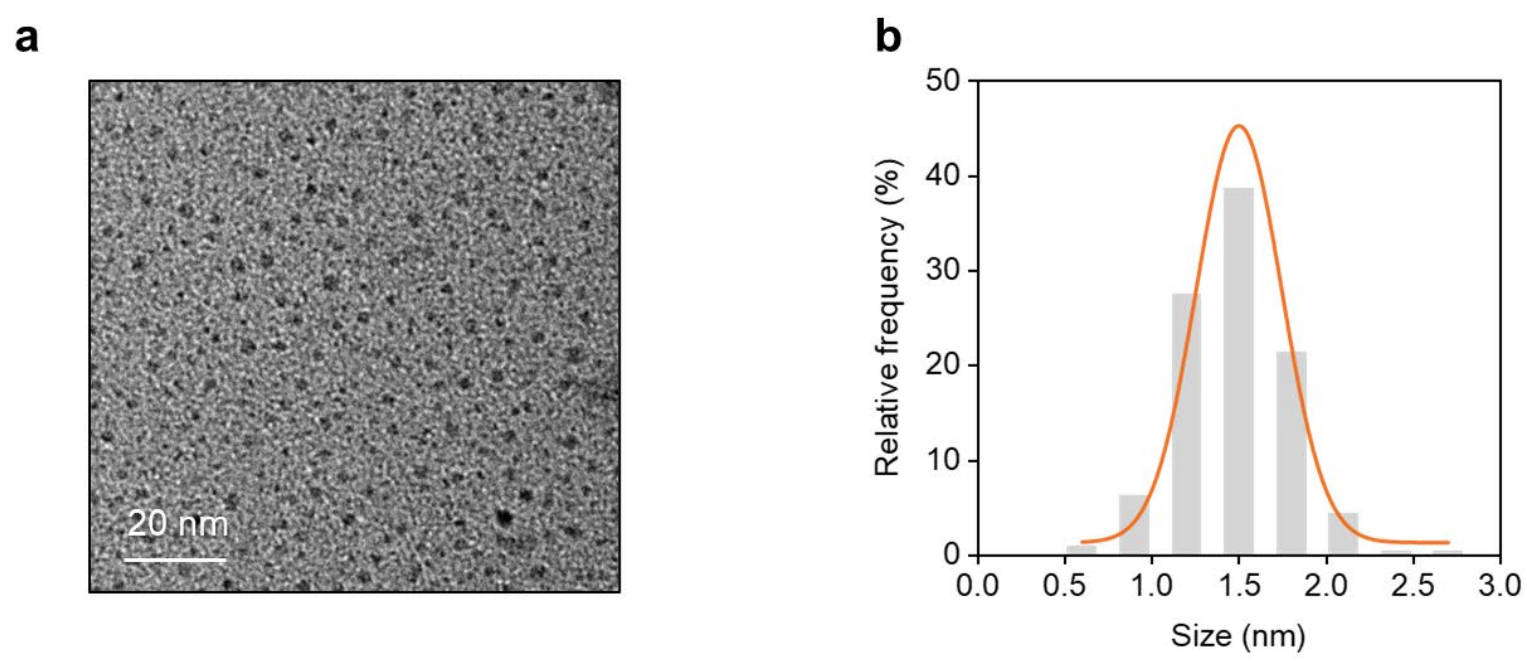

Figure S5. The transmission electron microscopy image (a) and size distribution (b) of calcium phosphate oligomers CPO.

7. Comparation between of GelMA-CPO scaffold and GelMA scaffold

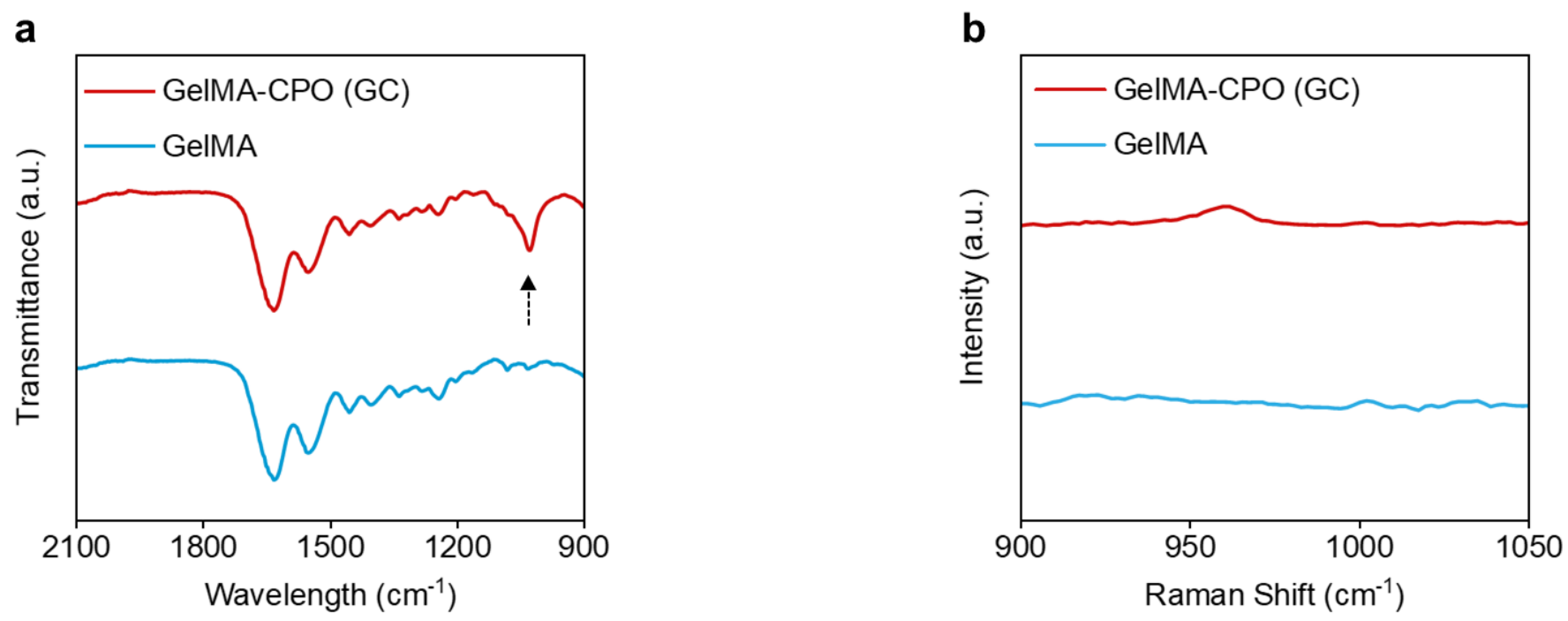

Figure S6. Fourier transform infrared spectra (a) and Raman spectra (b) of GelMA-CPO and GelMA.

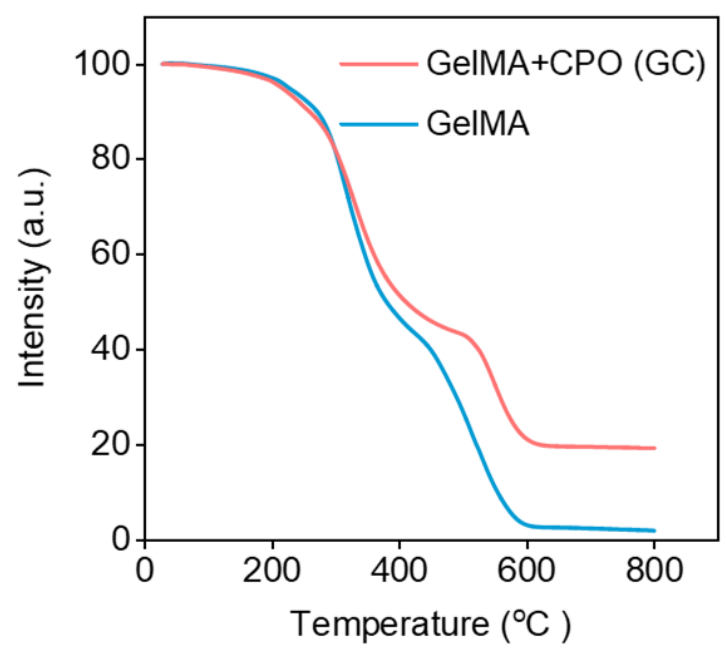

Figure S7. Thermogravimetry analysis of GelMA-CPO and GelMA with different thermal decomposition temperature.

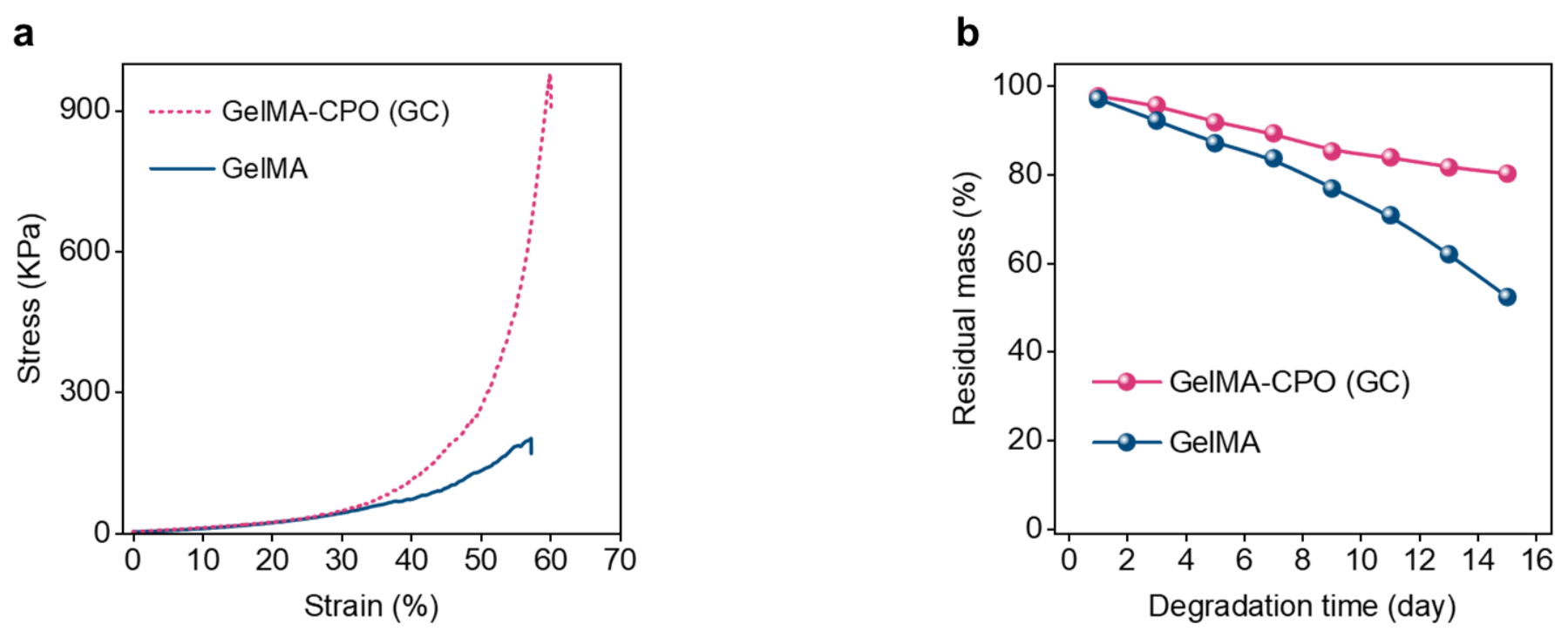

Figure S8. (a) Stress-strain curves of GelMA and GelMA-CPO. (b) Degradation curve of GelMA-CPO and GelMA.

8. Photothermal penetration depth comparison of different scaffolds

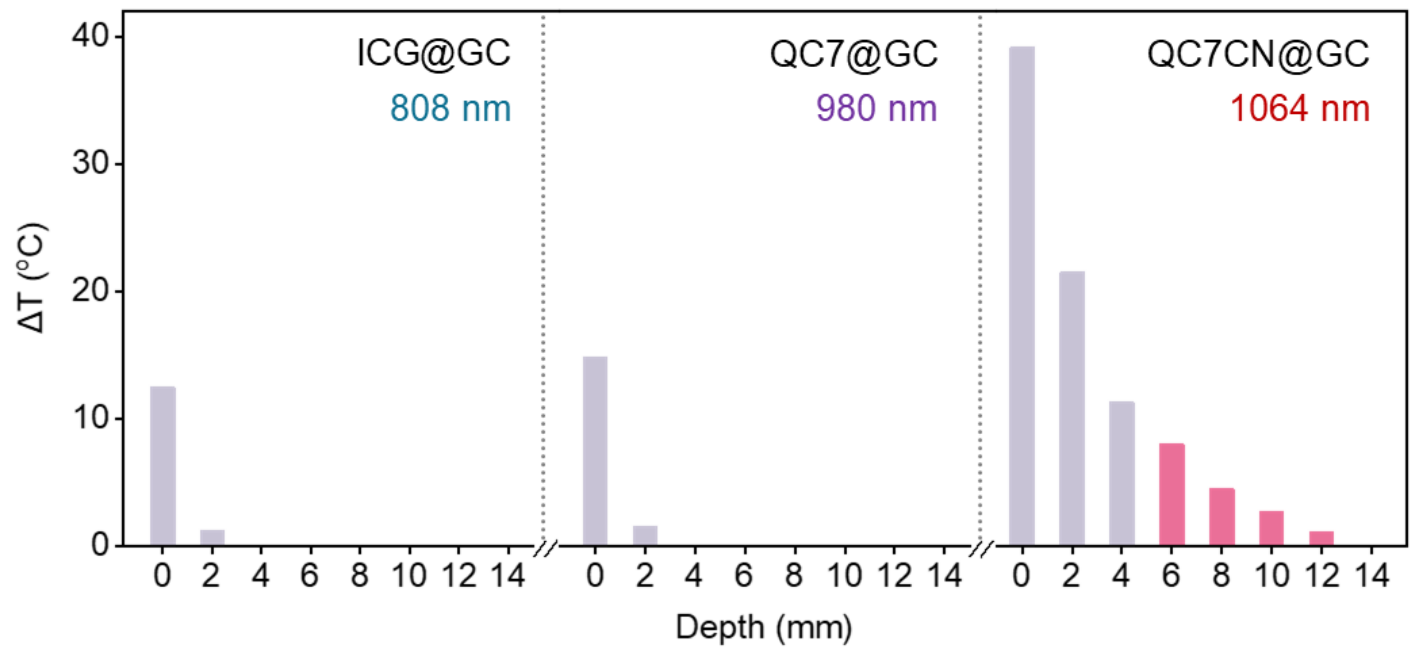

Figure S9. Photothermal penetration depth comparison among commercial ICG@GC (808 nm), QC7@GC (980 nm), and QC7CN@GC (1064 nm) under respective MPE conditions.

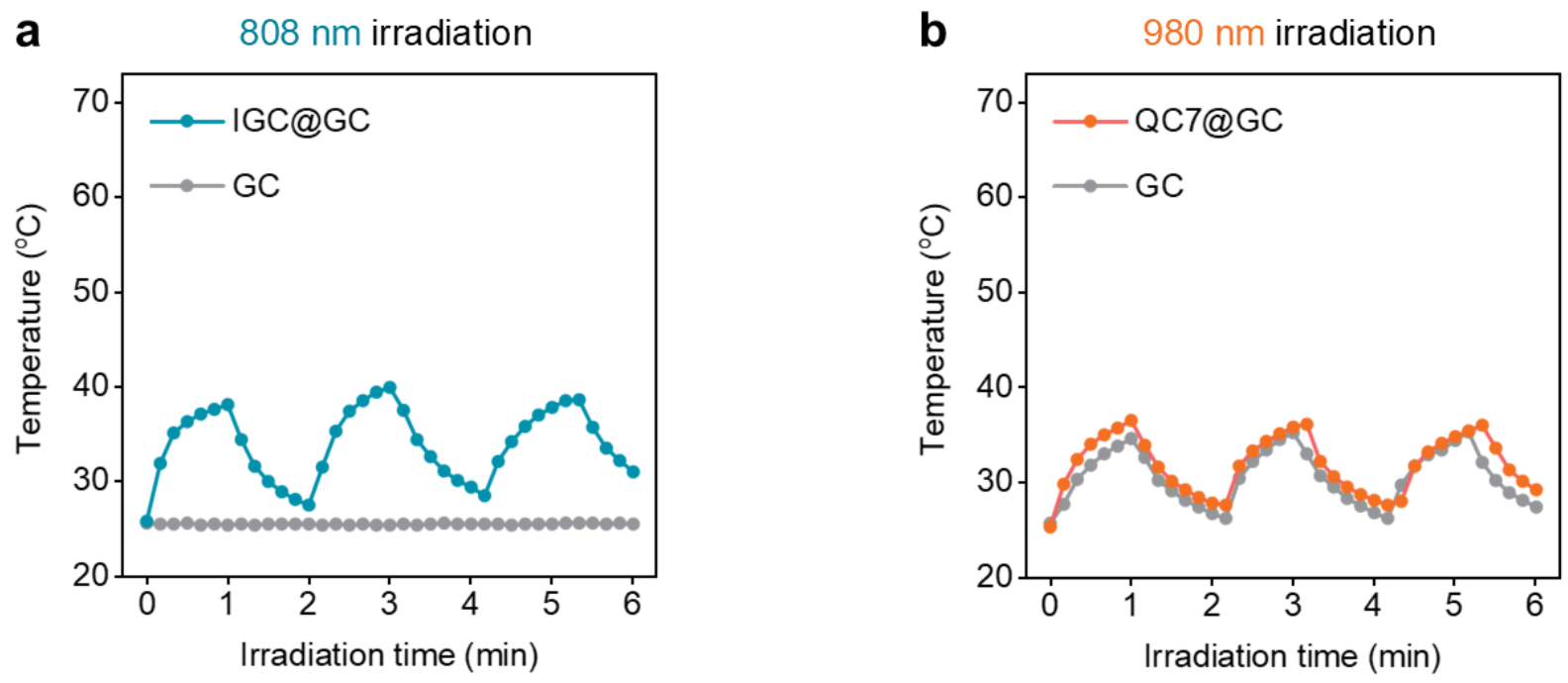

Figure S10. Photothermal potency of ICG@GC (a), QC7@GC (b) at their respective laser wavelengths and MPE-compliant power densities. we conducted a comparative analysis of scaffolds incorporating different dyes (ICG@GC, QC7@GC, and QC7CN@GC) at their matchable laser wavelengths and MPE-compliant power densities. Both ICG@GC and QC7@GC exhibited suboptimal photothermal performance, with maximum temperatures below 40 °C.

9. Characterization of thermosensitive copolymer (PNAm) and microsphere (MS)

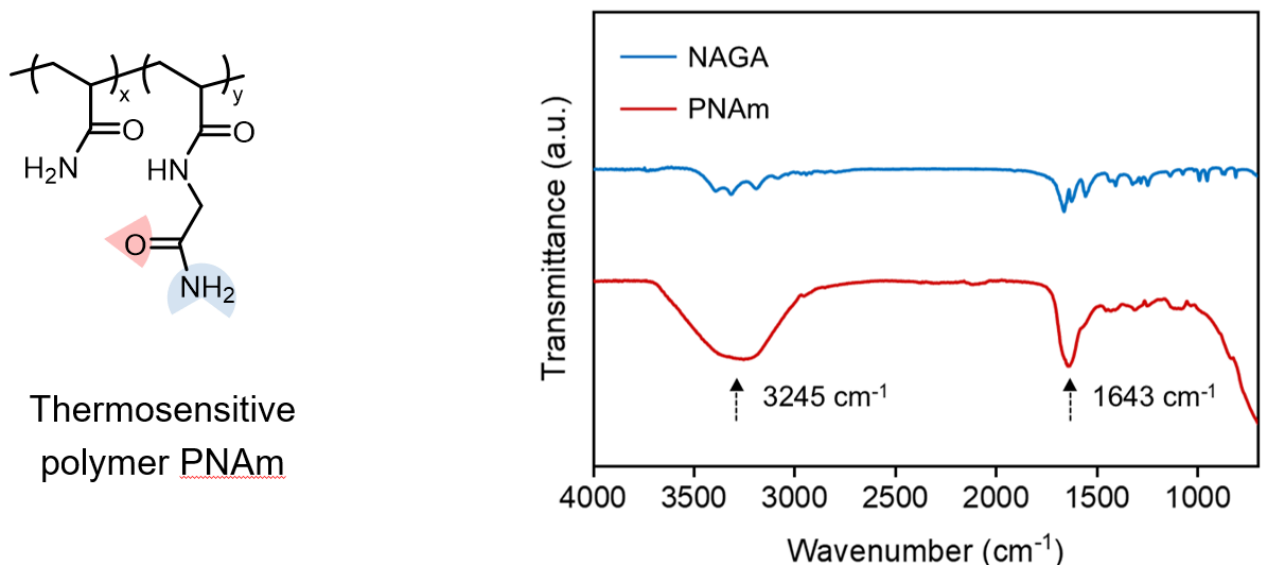

Figure S11. Fourier transform infrared spectra of NAGA monomer and PNAm copolymer.

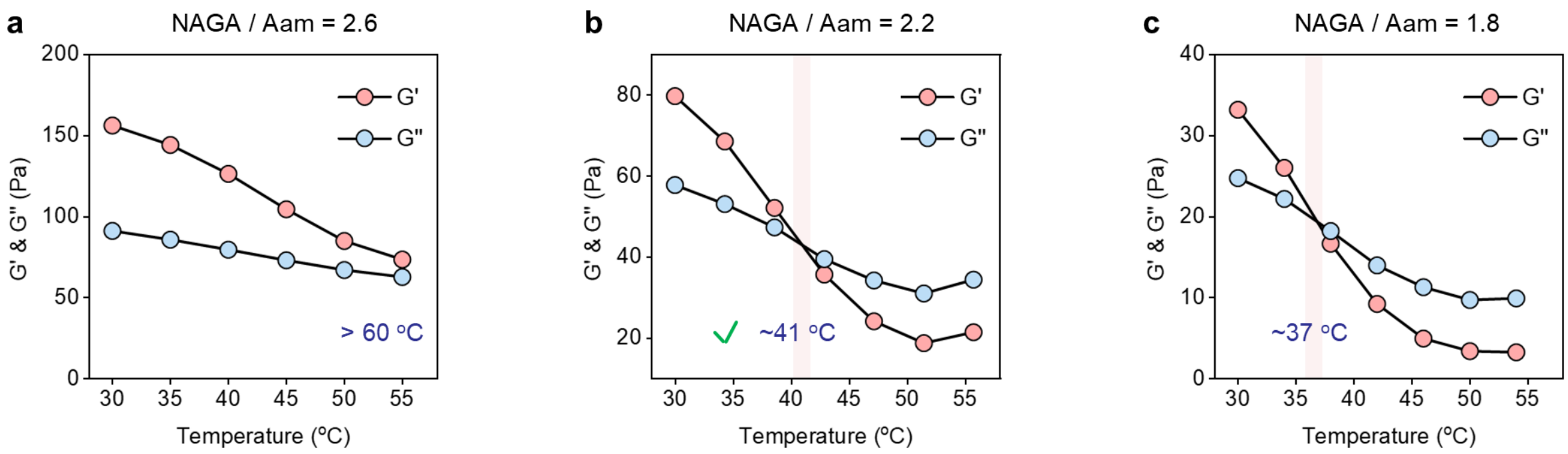

Figure S12. Temperaturedependence of storage modulus (G′) and loss modulus(G″) for polymer with varying ratios.

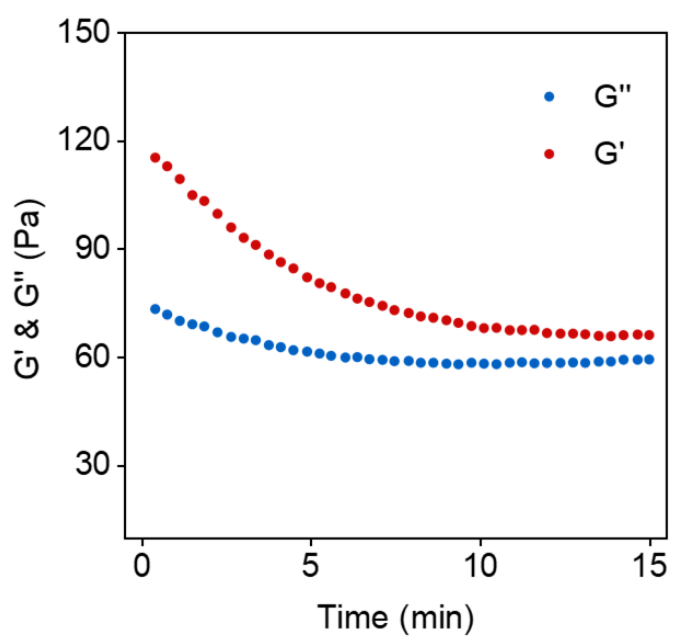

Figure S13. Storage modulus (G′) and loss modulus(G″) of PNAm under physiological conditions (37 °C).

10. Photothermal-triggered PTH release of QC7CN+PMS@GC scaffolds at different PMS loading

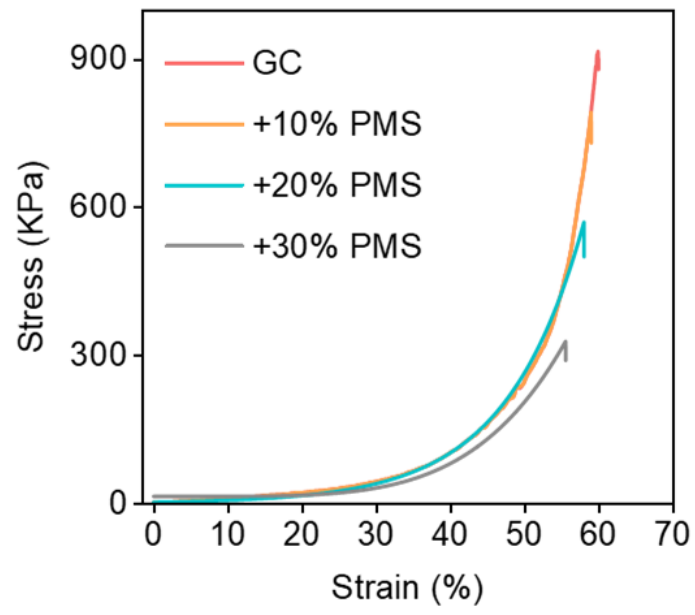

Figure S14. Stress-strain curves of scaffolds with varying PMS loading.

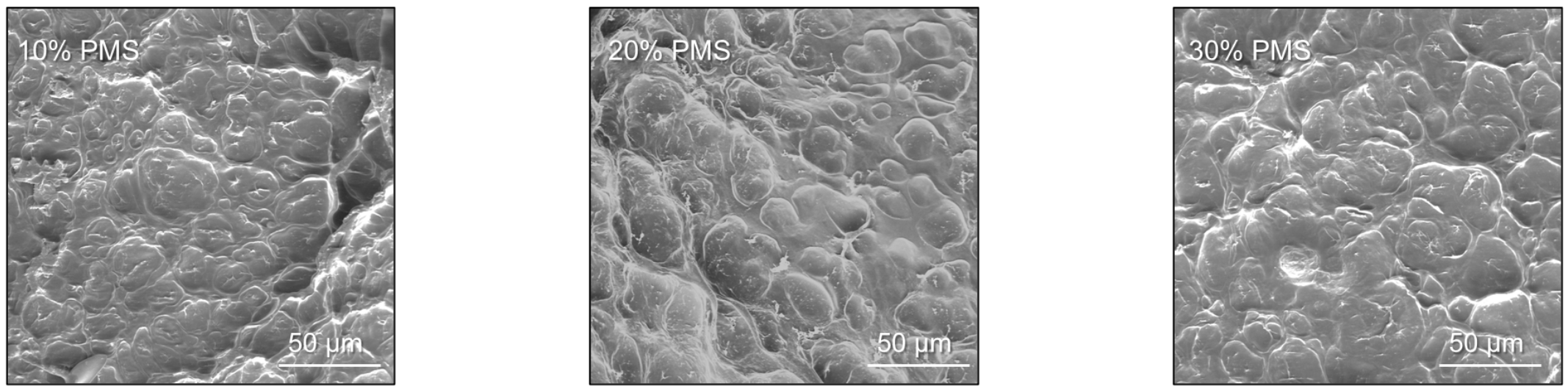

Figure S15. Scanning electron microscopy images of QC7CN+PMS@GC scaffolds with varying PMS loading.

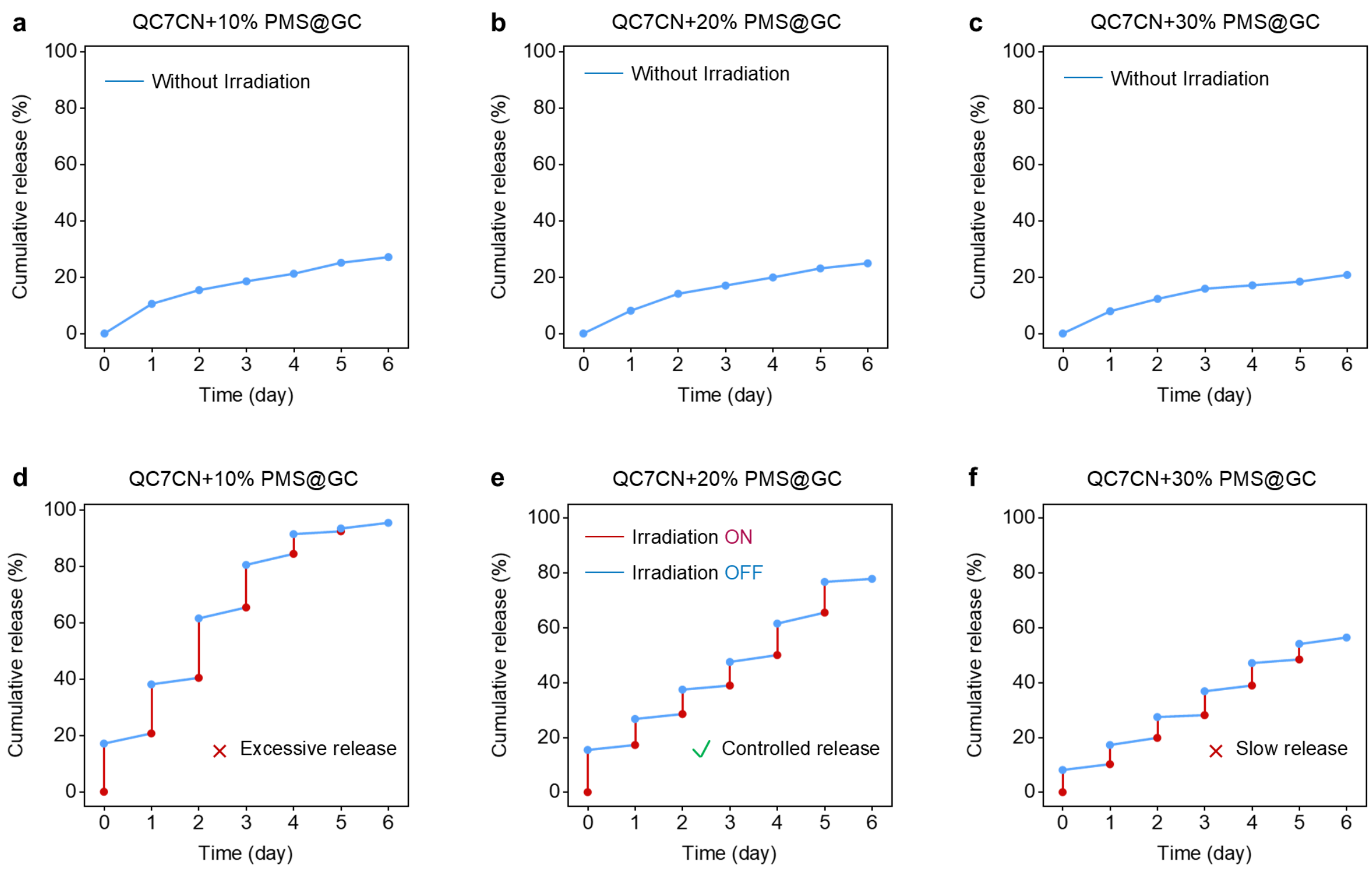

Figure S16. (a-c) PTH release of QC7CN+PMS@GC scaffolds at different PMS loading without irradiation. (d-f) Photothermal-triggered PTH of QC7CN+PMS@GC scaffolds at different PMS loading. All tests were conducted under 5 mm chicken breast tissue.

11. PTH stability after photothermal treatment

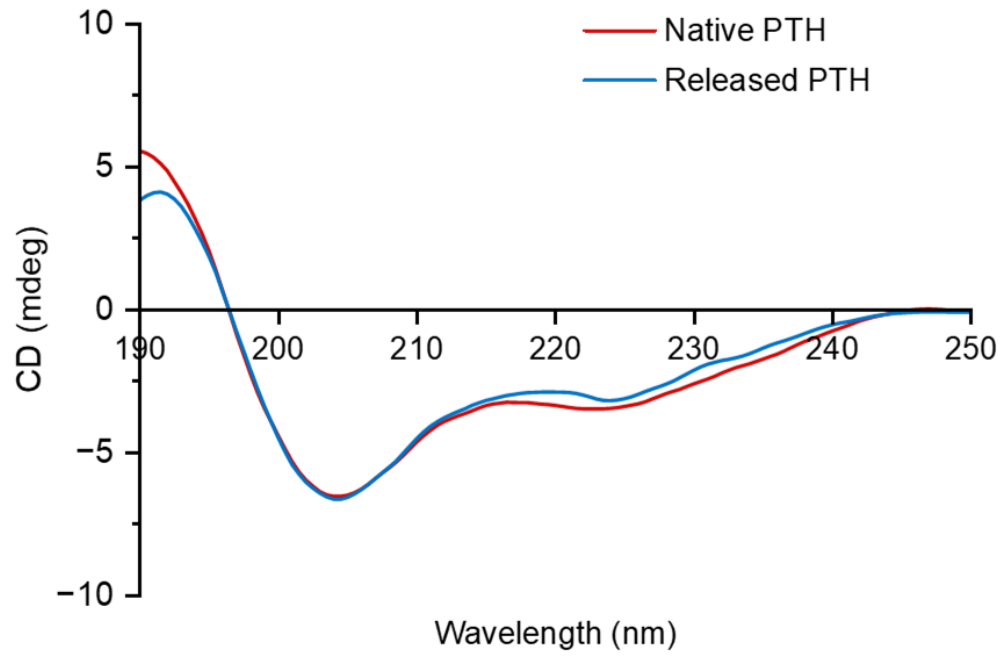

Figure S17. Circular dichroism spectra of native PTH and released PTH.

12. Biocompatibility of photothermal-responsive scaffolds

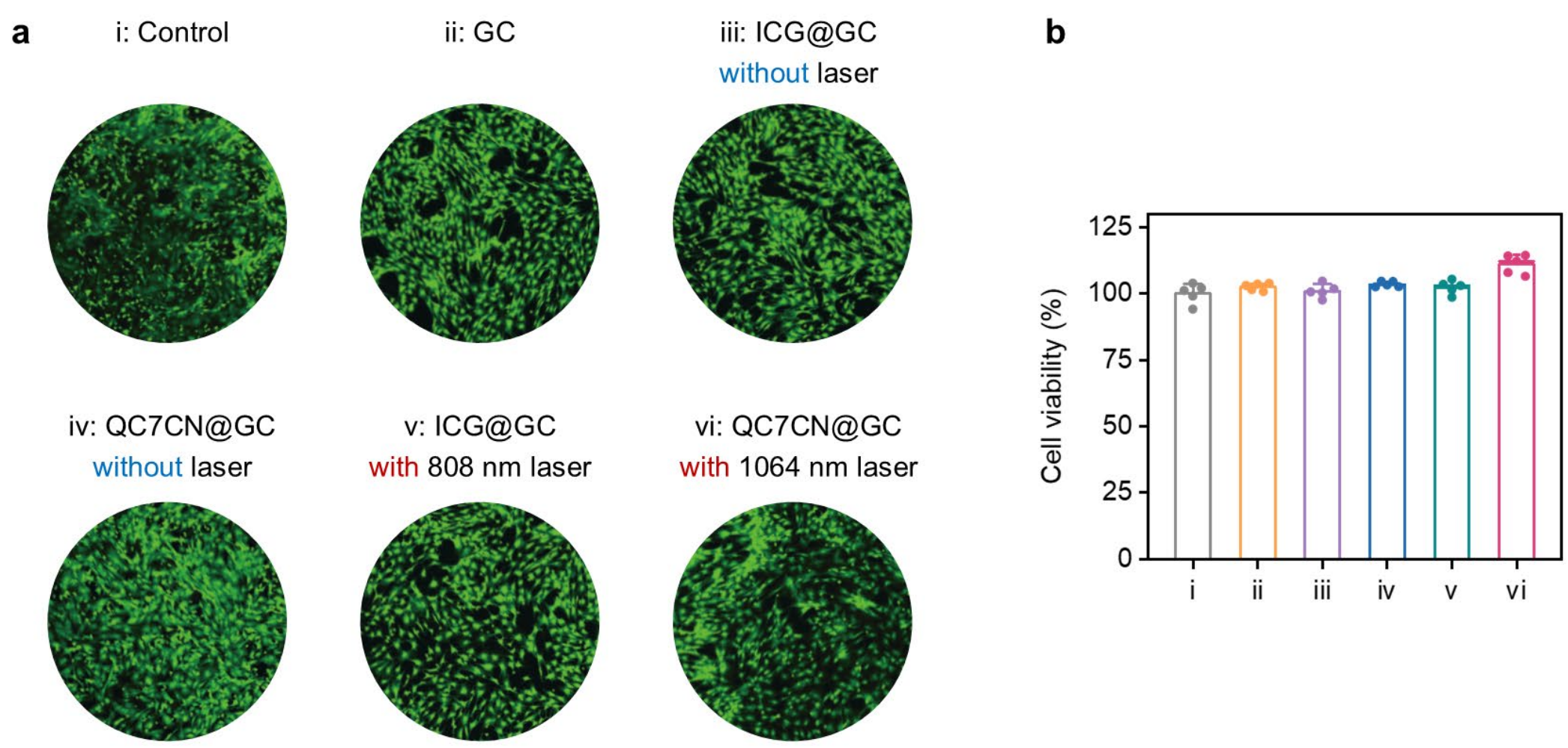

Figure S18. (a) Live and dead staining of BMSCs by different treatment. (b) Cell viability of BMSCs by different treatment.

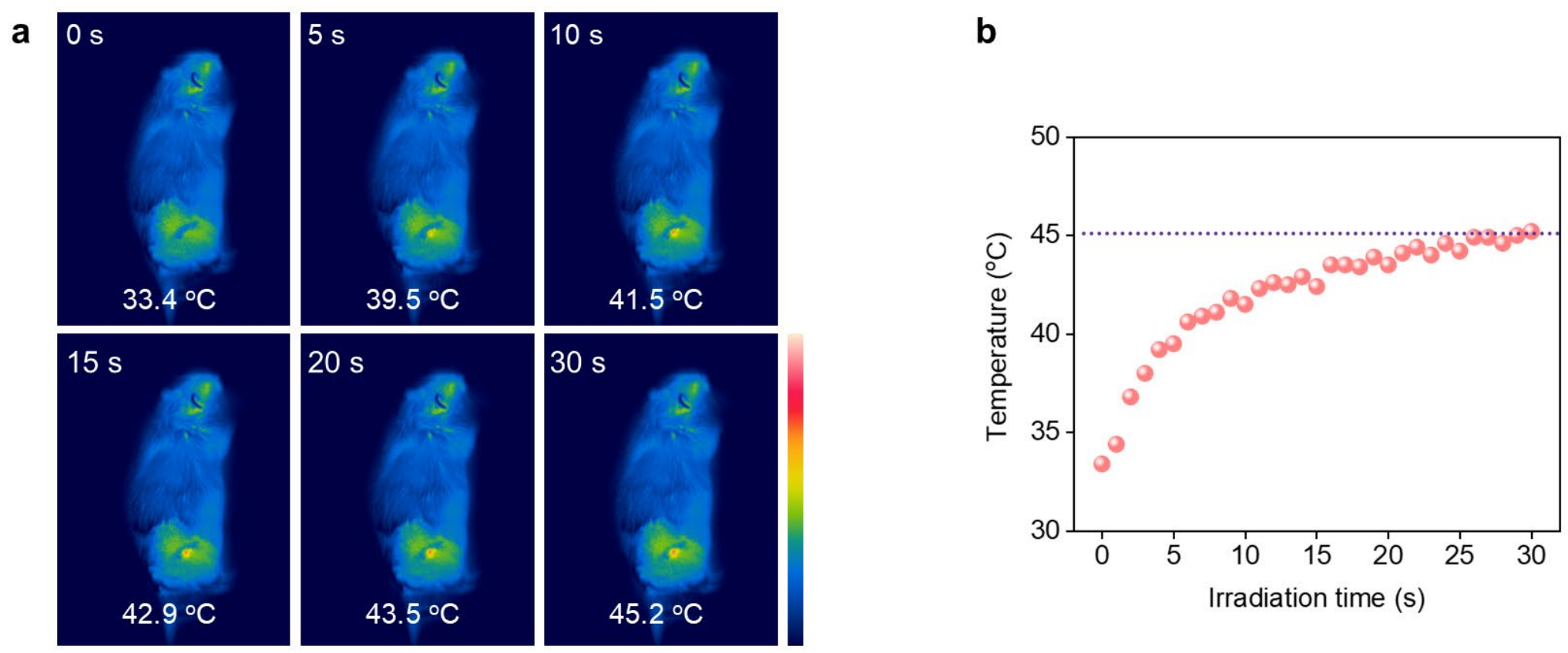

Figure S19. Photothermal images (a) and photothermal potency (b) of QC7CN+PMS@GC upon 1064 nm irradiation.

13. Photo-triggered pulsatile PTH release for osteogenic differentiation

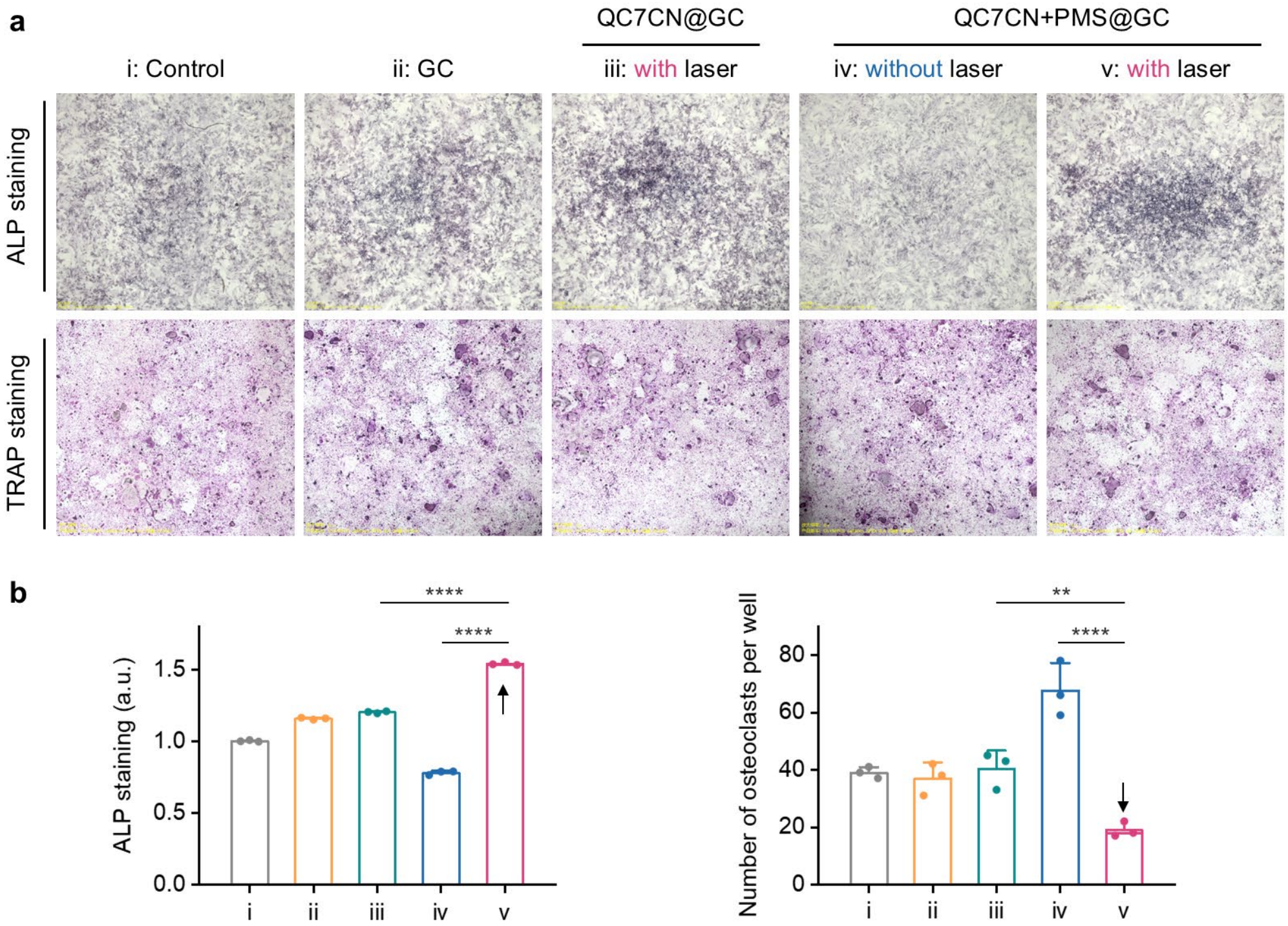

Figure S20. (a) ALP staining of BMSCs after co-culture in an osteogenic matrix for 7 days, and TRAP staining of osteoclasts after different treatments for 3 days. (b) Quantitative analysis of ALP/TRAP staining.

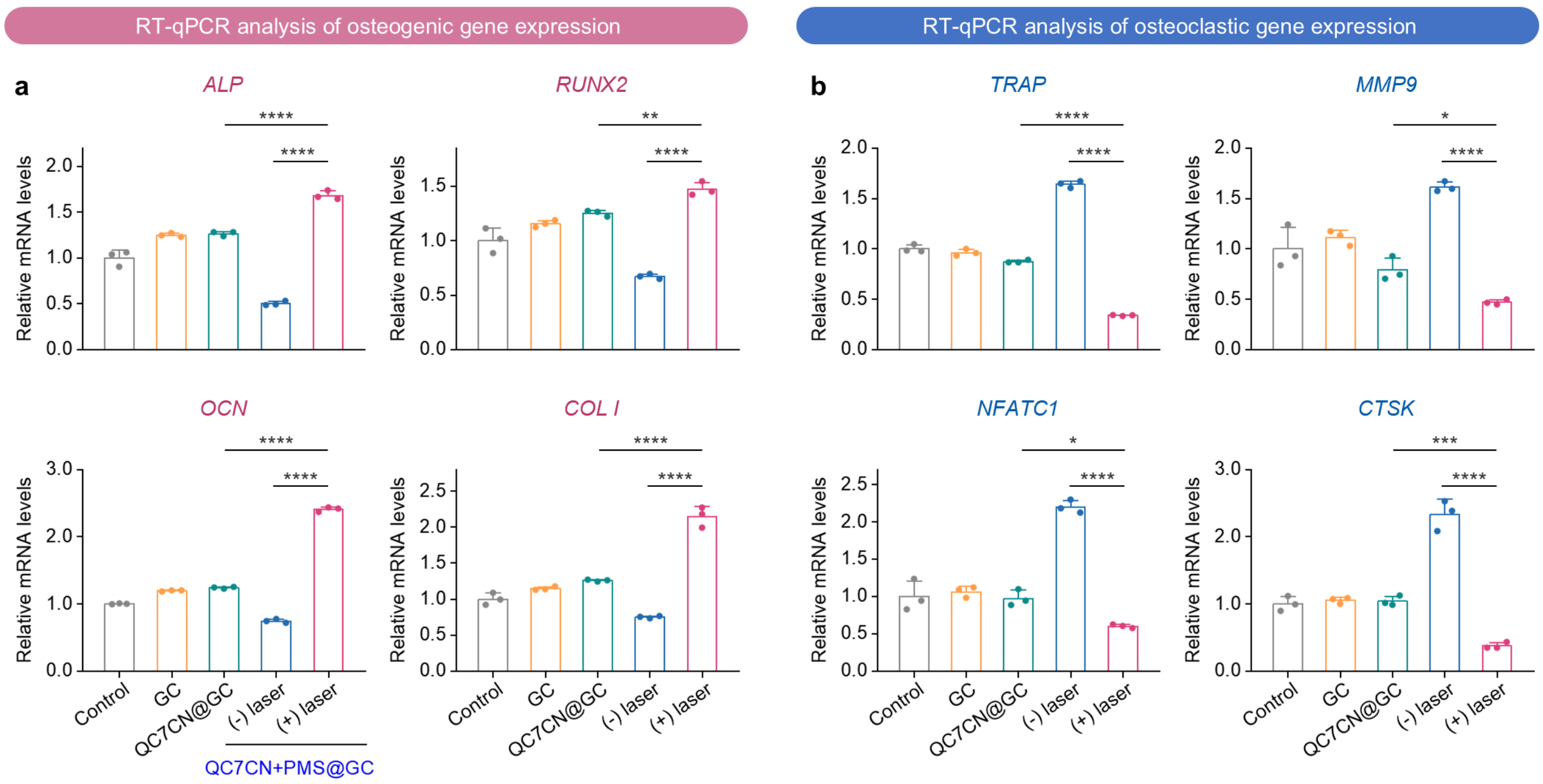

Figure S21. (a) RT-qPCR analysis of osteogenic marker genes (ALP, RUNX2, OCN, and COL I) in BMSCs after different treatments. (b) RT-qPCR analysis of osteoclastic marker genes (TRAP, MMP9, NFATC1, and CTSK) in osteoclasts after different treatments. Results showed that photothermal stimulation alone had negligible effects on differentiation. Notably, "QC7CN+PMS@GC without laser" group, with passive PTH leakage, induced obvious osteoclastogenesis, whereas laser-triggered pulsatile release significantly promoted osteogenesis.

14. PCR primer sequences

Table S1. PCR primer sequences

Target gene	Forward primer sequence (5'-3')	Reverse primer sequence (5'-3')
ALP	TGGAACACTGGGTCCCATA	GACCTGGTCTTCCCTCCAA
RUNX2	ATCCAGCCACCTTCACTTACACC	GGGACCATTGGGAACTGATAGG
OCN	GCCCTGACTGCATTCTGCCTCT	TCACCACCTTACTGCCCTCCTG
COL I	TGGATGGCTGCACGAGT	TTGGGATGGAGGGAGTTTA
NFATC1	GTGGCAGCCATCAACGCCCT	TACGAGGCCTGTGGCACCGA
TRAP	ATGAGAACGGTGTGGGCTA	GAGCCTATCTCCACGTATGT
CTSK	ACTGCGACCGTGATAATGTGAAC	TCCGAGCCAAGAGAACATAGCC
Mmp9	TTCAAGGACGGTCGGTATT	CTCTGAGCCTAGACCCAACTTA
Gapdh	ACCCTCAACAGGGATGCTTA	GATACGGCCAAATCCGTTCA

15. Alizarin red S (ARS) staining about osteogenic differentiation

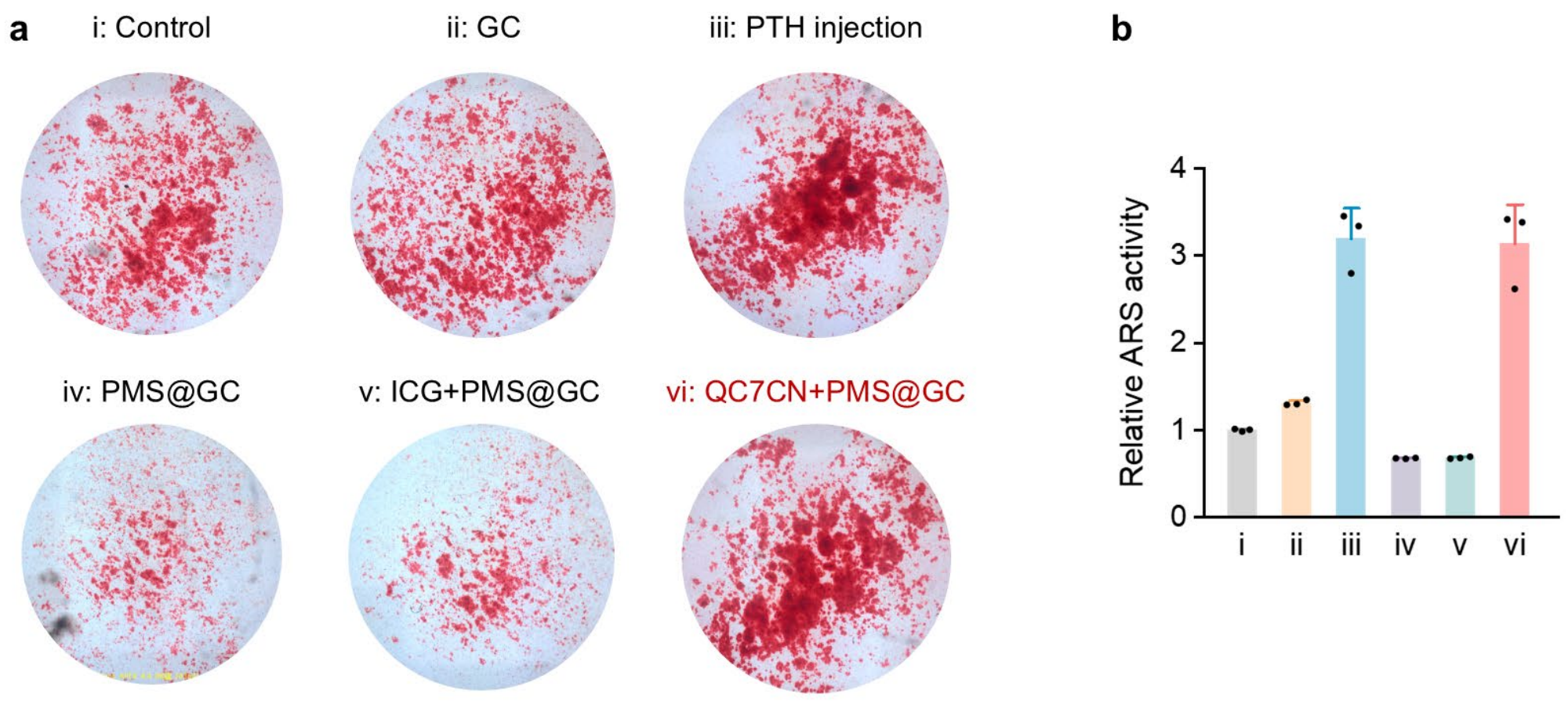

Figure S22. Alizarin red S (ARS) staining and quantitative analysis of BMSCs in an osteogenic matrix for 21 days.

16. Deep-tissue pulsatile PTH delivery for osteoporotic bone repair

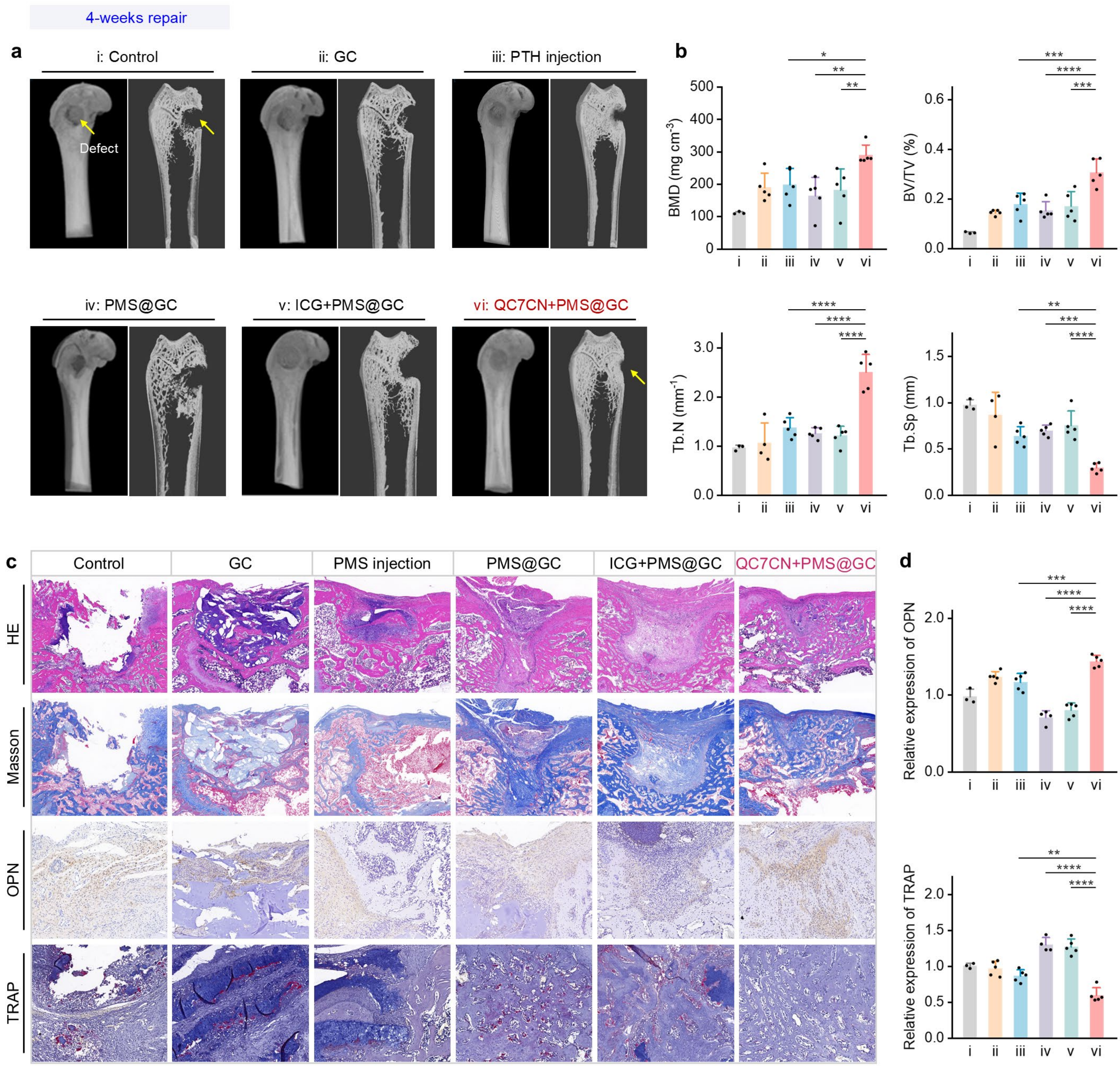

Figure S23. (a) Representative 3D reconstructed micro-CT images across different treatment groups at 4 weeks. (b) Quantification of bone mineral density (BMD), bone volume fraction (BV/TV), trabecular number (Tb.N), trabecular separation (Tb.Sp) at week 4. (c) HE and Masson staining, and immunohistochemical staining for osteopontin (OPN, osteogenesis marker) and TRAP (osteoclastogenesis marker) of femoral defect sections. (d) Quantitative analysis of OPN expression and TRAP expression at week 4. Statistical significance: *$p < 0.05$, **$p < 0.01$, ***$p < 0.001$, ****$p < 0.0001$; ns, not significant.
